# Psychosocial Interventions in Cancer Survivorship: A Rapid Scoping Review of the Recent PubMed-Indexed Literature

**DOI:** 10.3390/cancers18203385

**Published:** 2026-10-20

**Authors:** Anita Müller, Selma Barbot, Morgan Dondin, Clotilde Potez, Sylvie Dolbeault, Anne Brédart, Maria Die Trill, Bernhard Holzner

**Affiliations:** 1 Psycho-Oncology Unit, Supportive Care Department, Institut Curie, 75005 Paris, France; 2 Social Sciences and Humanities, and Outcome Research (SHARE Team), Translational Research Department, PSL Research University, Institut Curie, 75005 Paris, France; 3 Psychopathology and Health Process Laboratory UR4057 ED 261, Psychology Institute, Paris City University, 92774 Boulogne-Billancourt, France; 4 VCR, Ecole de Psychologues Praticiens de L’institut Catholique de Paris—Équipe D’accueil Religion, Culture et Société, 75006 Paris, France; 5 ER IPC-Facultés Libres de Philosophie et de Psychologie, 70 Avenue Denfert-Rochereau, 75014 Paris, France; 6 Laboratoire Psychopathologie et Processus de Changement, EA 2027, Université Paris 8, 2 Rue de la Liberté, 93200 Saint-Denis, France; 7 Research Centre in Epidemiology and Population Health (CESP), INSERM U1018, Paris Saclay University, 94800 Villejuif, France; 8 Psycho Oncology Unit, Madrid Site Cancer Center, Clínica Universidad de Navarra, 28027 Madrid, Spain; 9 University Hospital of Psychiatry II, Medical University of Innsbruck, 6020 Innsbruck, Austria; bernhard.holzner@tirol-kliniken.at

**Keywords:** psychosocial intervention, cancer survivorship, distress, fear of cancer recurrence, eHealth

## Abstract

**Background/Objectives**: In recent years, the number of cancer survivors has increased. Psychological difficulties or disorders are frequent during survivorship (e.g., fear of cancer recurrence, anxiety, depression, and insomnia). Various interventions have been developed to address survivors’ specific psychosocial needs. This rapid scoping review aimed to define the interventions that have been developed for adult cancer survivors by describing (1) the type of intervention, (2) their measured psychosocial outcome variables, and (3) participants’ main characteristics. **Methods**: PubMed was searched for articles published between 2020 and 2025. The search strategy combined terms related to cancer survivorship, psychosocial outcomes, and interventions. Titles and abstracts were screened, and data were extracted using a structured form specifying study type, population, intervention, and outcome characteristics. PRISMA-ScR guidelines were followed. **Results**: Out of 1743 screened studies, 80 were included (67 studies, 13 systematic reviews/meta-analyses), representing approximately 7880 cancer survivors (from the n = 67). Nearly 45% of included studies were conducted in the United States, and most interventions were developed for breast cancer survivors (38.8%) or included a majority of female participants. Physical activity (22.39%), psychoeducation (17.9%), and cognitive behavioural therapy (16.4%) were the most common intervention types. About a third incorporated eHealth or telehealth. Two-thirds of studies (65.7%) reported predominantly positive effects on psychosocial outcomes. Few interventions included cultural adaptations and targeted minority or socioeconomically disadvantaged populations. **Conclusions**: Most interventions for cancer survivors targeted breast cancer, female survivors in high-income countries. Developing and implementing culturally adapted and cost-efficient interventions, particularly for underrepresented survivor groups, remains a priority for future research and implementation.

## Simple Summary

In recent years, the number of cancer survivors has increased significantly. Psychological difficulties or disorders are frequent during survivorship, such as fear of cancer recurrence, anxiety, depression, and insomnia. Various interventions have been developed to address survivors’ specific psychosocial needs. The present review aimed to identify these interventions, as well as measured psychosocial outcome variables and participants’ main characteristics. Therefore, a rapid scoping review was conducted on PubMed, identifying 1743 studies; after title, abstract, and full-text screening, 67 studies and 13 systematic reviews or meta-analyses were included. Despite a high number of developed interventions, around 40% of them have been developed targeting breast cancer survivors. Even when various cancer types were included, a majority of participants were female and/or breast cancer survivors. Few interventions included cultural adaptations or adaptations for minority population groups. Developing and implementing culturally adapted interventions remains a priority.

## 1. Introduction

Over the last decades, the number of cancer survivors has continuously increased. The World Health Organization (WHO) estimated there were 53.5 million cancer survivors in 2022 [1]. This increase may be attributed to two main factors: (1) an increase in cancer diagnoses, whether by early detection and specific screening programmes or by environmental factors (e.g., smoking, sun exposure) and an overall ageing population; (2) medical breakthroughs (e.g., development of new treatments, such as immunotherapies and targeted therapies) that enable more cancer patients to live beyond former life expectancies [2].

With an increasing number of cancer survivors comes a growing need to understand their specific psychosocial needs and difficulties (e.g., fear of cancer recurrence (FCR), post-traumatic stress symptoms, or distress related to life changes such as persisting symptoms, altered body image, existential distress, social isolation or lack of support, or employment or other financial concerns). Since the middle of the 80s, several reports, guidelines, and programmes have been published [2]. This builds on earlier work addressing patients’ professional rehabilitation after cancer treatment [3]. Yet, despite nearly four decades of progress, the field is still continuously progressing while improving the foundations, such as the term “survivorship”, the meaning of which is still under discussion. In North American publications, “survivorship” was originally considered an integrative concept encompassing various disease stages. More precisely, Mullan distinguished “acute survival” (diagnosis and treatment phases), followed by “extended survival” and, lastly, “permanent survival” [4]. This model was subsequently revised with the addition of a “transitional survivorship” phase following primary treatment. This phase ranges from the first months after treatment up to one year, before entering “extended survivorship”, which includes patients living with cancer as a chronic disease or those in remission. Finally, “permanent survivors” encompass individuals who continue to experience any psychological or physical side effects or long-term consequences of cancer and/or its treatments [5]. More recently, the current literature considers cancer survivorship from the time of diagnosis throughout the lifespan [6]. These successive definitions of survivorship have contributed to conceptualising the diverse trajectories of people living with and beyond cancer and to identifying survivors’ specific difficulties, needs and quality of life (QoL) [7]. Recent research on cancer survivors has shown that even though some patients agree with this term and perceive it as a positive identity, others feel “offended” by it or “rejected” it [8]. According to the latter, this term does not accurately represent the full extent of what one can lose to cancer and, consequently, fails to capture the considerable and (for many) irreversible impacts that cancer can have on various domains of life [9]. This is a crucial consideration, considering that patients’ survivor identity can have an impact on their overall QoL, more precisely, the way they define themselves [10].

Cancer-related fatigue, pain, physical limitations, and severe and/or lasting cognitive impairments can significantly impact survivors’ daily and professional life. Depending on the treatment, sequelae can vary in type, intensity, and duration [11]. Unemployment rates tend to be higher among cancer survivors compared to the general population, particularly among female cancer survivors, or those with a physically demanding job, requiring, for example, physical or manual labour [12]. This is a critical issue in cancer survivorship, as it can have significant financial and social consequences [13]. These consequences also shape the motivation to return to work, which is usually driven by a desire for normalcy or financial necessity [14].

Cancer and cancer survivorship can broadly affect interpersonal relations, as well as the social environment in general [15]. Although a cancer diagnosis and its treatment may provide an opportunity for personal or relational growth, restructuring and a redefinition of values, this is not consistently observed amongst every patient [16,17]. A negative impact on social wellbeing is more frequently reported among survivors who are either younger, female, or with a lower income [18]. In the presence of persistent side effects, family members are often required to maintain a caregiver role beyond the end of treatment. This can sometimes lead to frustration, guilt, or helplessness [19]. Furthermore, relatives’ expectations of a rapid and complete recovery, or even a return to “life before cancer”, can generate tension within family systems. Support provided by social networks may decline after the end of treatment [20]. Both survivors and relatives may experience loneliness, lack of support, or invalidation as they feel that their emotions and needs are misunderstood [21]. Within families, caregivers’ unmet needs can lead to exhaustion and eventually caregiver burnout [22]. Survivors may feel abandoned and reluctant to reengage in social activities in this context, while also feeling stuck in a dependency dynamic sometimes [20]. Additionally, difficulties regarding body image and sexual functioning can negatively impact romantic relationships and intimacy [23]. This emphasises the importance of addressing survivorship as a multidimensional model requiring supportive interventions on individual and family levels.

Psychiatric disorders can arise at any time between initial diagnosis and years into “permanent survivorship”. Ten to 34% of cancer survivors suffer from specific psychiatric disorders, such as delirium, anxiety, major depression, or adjustment disorders [24]. Furthermore, a high percentage of cancer survivors experience FCR, representing the most common long-term psychological sequelae of the disease and its treatments [25]. Such psychosocial disorders can manifest either individually or concurrently, in which case they are described as a “symptom cluster”. Clinically speaking, experiencing pain during a prolonged period can be exhausting and is often associated with anxiety or depressive symptoms. Various interventions have been developed in order to address these symptoms or symptom clusters [26], such as physical activity (PA), psycho-education (PE), different components used in cognitive and behavioural therapy (CBT), and more.

Existing reviews on psychosocial interventions for cancer survivors frequently focus on a single cancer type [27], a single intervention modality [28,29], or a specific issue of survivorship (e.g., fatigue), including mainly RCTs. Given the rapid development in this field, an updated, broader synthesis is needed to fill the present gap in cross-diagnosis, cross-intervention overviews. According to the American Psychosocial Oncology Society (APOS, 2020), psychosocial interventions address the emotional, social, practical, and behavioural consequences of cancer and its treatment during survivorship. The present rapid scoping review (ScR) aims to address this gap by providing an updated, broad map of psychosocial interventions developed for adult cancer survivors across cancer types. Its specific objectives are (1) to describe the range of these interventions; (2) to identify the psychosocial outcomes they assess; and (3) to describe participants’ main characteristics, including the cancer populations that are most commonly targeted.

## 2. Materials and Methods

A rapid ScR was conducted to map out the range and characteristics of psychosocial interventions available for adult cancer survivors. ScR aims to identify the extent and nature of the literature on a topic, clarify key concepts, and identify knowledge gaps [30]. A rapid ScR was considered appropriate as the objective was to provide a broad overview of interventions, population types and outcomes. Five methodological steps were followed: (1) identification of the research question, (2) identification of relevant studies, (3) selecting studies, (4) charting data, (5) summarising and reporting results [31]. Rapid reviews mostly follow these steps while also enabling authors to accelerate the review process by shortcutting some steps [32,33]. For the present review, the only shortcut consisted of using only PubMed (MEDLINE). According to guidelines, this can be considered the main database [33] and was found to be appropriate regarding this review’s objectives, as a majority of health-psychology literature is indexed in PubMed [34]. The search was conducted using an algorithm that combined three concepts (1) cancer survivorship, including terms like (“cancer survivorship” [Title/Abstract] OR “cancer survivor” [Title/Abstract] OR “cancer surveillance” [Title/Abstract] OR “après-cancer” [Title/Abstract]), AND (2) psychosocial outcomes such as (“anxiety” OR “distress” OR “depression” OR “depressive symptoms” OR “psychological well-being” OR “psychological wellbeing” OR “sleep” OR “insomnia” OR “psychosocial difficult*” OR “quality of life”), AND (3) interventions (“intervention” OR “therapy” OR “eHealth” OR “e-health” OR “telehealth” OR “complex intervention” OR “blended intervention” OR “selfhelp” OR “self-help” OR “application”). The search was restricted to English, French, and German articles, and publication dates had to be between 2020 and 2025. Articles have been downloaded into Zotero and have been assessed for inclusion or exclusion based on Title and Abstract Screening. Experimental, cohort, or pilot studies, systematic reviews (SR), and meta-analyses (MA, with a focus on psychosocial interventions for adult cancer survivors, have been included, as long as they evaluated participants’ exposure to a defined psychosocial intervention (e.g., pre–post or intervention versus non-intervention comparisons), regardless of the cancer diagnosis (Supplementary File S2, Search Strategy). Observational cohort studies provide real-world evidence, which is increasingly important for health policies, outside the context of highly controlled trials. Therefore, their inclusion is interesting. However, observational cohorts describing psychosocial outcomes in survivors without an intervention component were excluded. Including both evidence synthesis (SR and MA) and original articles enables a comprehensive mapping of existing studies and already consolidated evidence. PE, PA, CBT, functional and multidisciplinary care, as well as psychological or self-directed support intervention types have been considered as long as reporting on at least one psychosocial endpoint was sufficiently detailed. To follow the aims of this review, approaches including alternative medicine have only been considered in combination with any of the previously cited intervention types. Feasibility studies were eligible for inclusion, as long as they reported detailed psychosocial outcomes. Studies without clear reporting of psychosocial outcomes, focusing on survivors aged less than 18 years old and/or without a cancer diagnosis, have been excluded. Articles on adult survivors of paediatric cancer have also been excluded. Furthermore, umbrella reviews and case studies have been excluded. Full texts were retrieved whenever exclusion could not be determined based on title or abstract. Data were extracted from all included articles using a data extraction form including characteristics of the study, study population, intervention type and category, psychosocial measures used, as well as main outcomes. Articles were divided between two reviewers (AM, SB) for screening and data extraction. Disagreements were systematically resolved through discussion until consensus was reached. To further support consistency, a subset of articles was additionally screened and extracted independently by both reviewers, with no major discrepancies identified. Consistent with the objectives of a rapid ScR, no formal risk-of-bias assessment was conducted. The protocol of this review has not been registered, and therefore no registration number can be provided. Summarised data are reported in frequencies and percentages. This rapid ScR is reported in accordance with the PRISMA guidelines and specifically the extension PRISMA-ScR for scoping reviews [35,36] (Supplementary File S1, PRISMA-ScR checklist). Tables with detailed results are reported (Supplementary File S3, Tables S1 and S2).

## 3. Results

**Figure 1:**
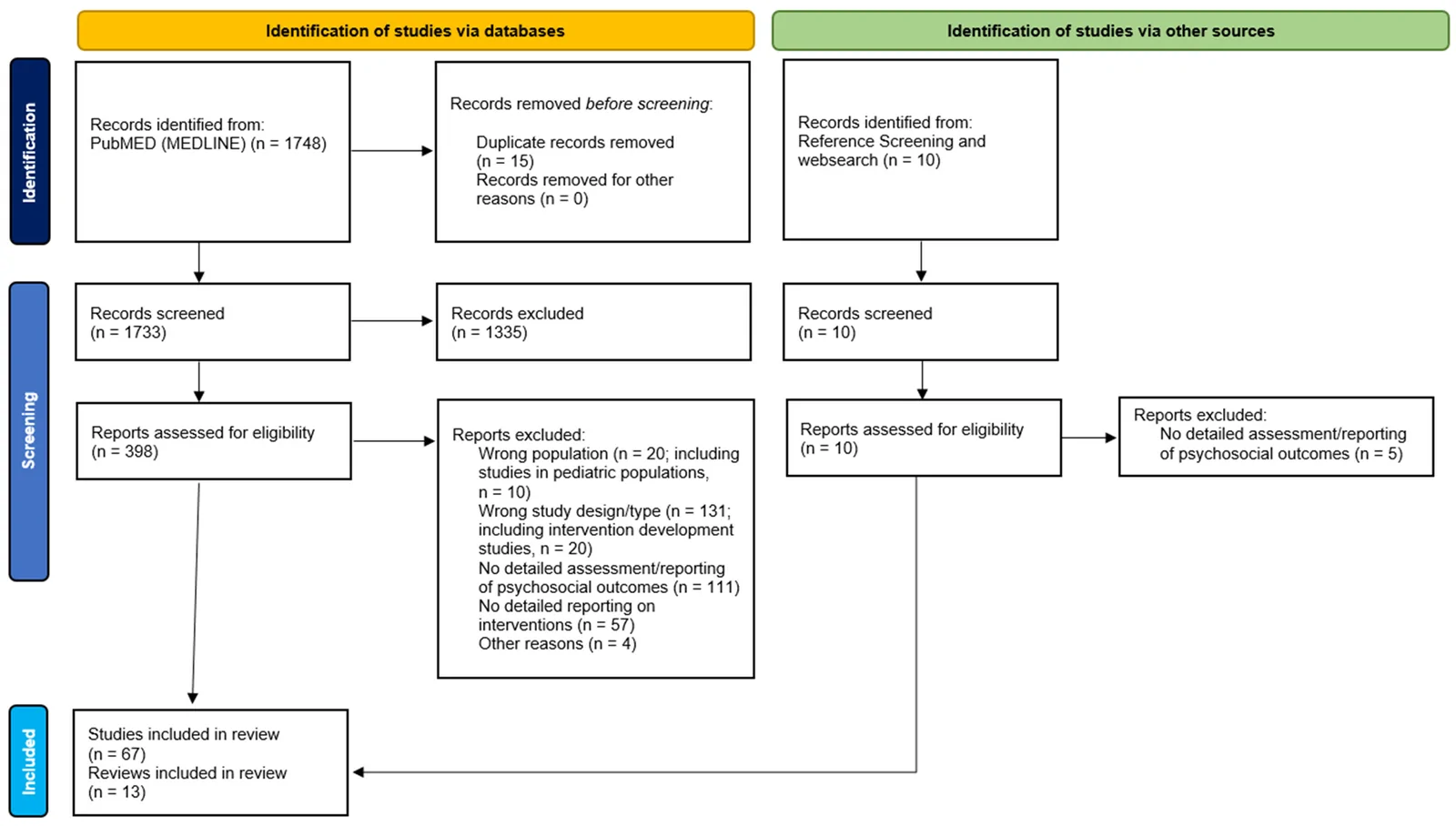
Flow chart of the review process. It illustrates identified, screened, and included studies, with reasons for exclusion.

**Figure 2:**
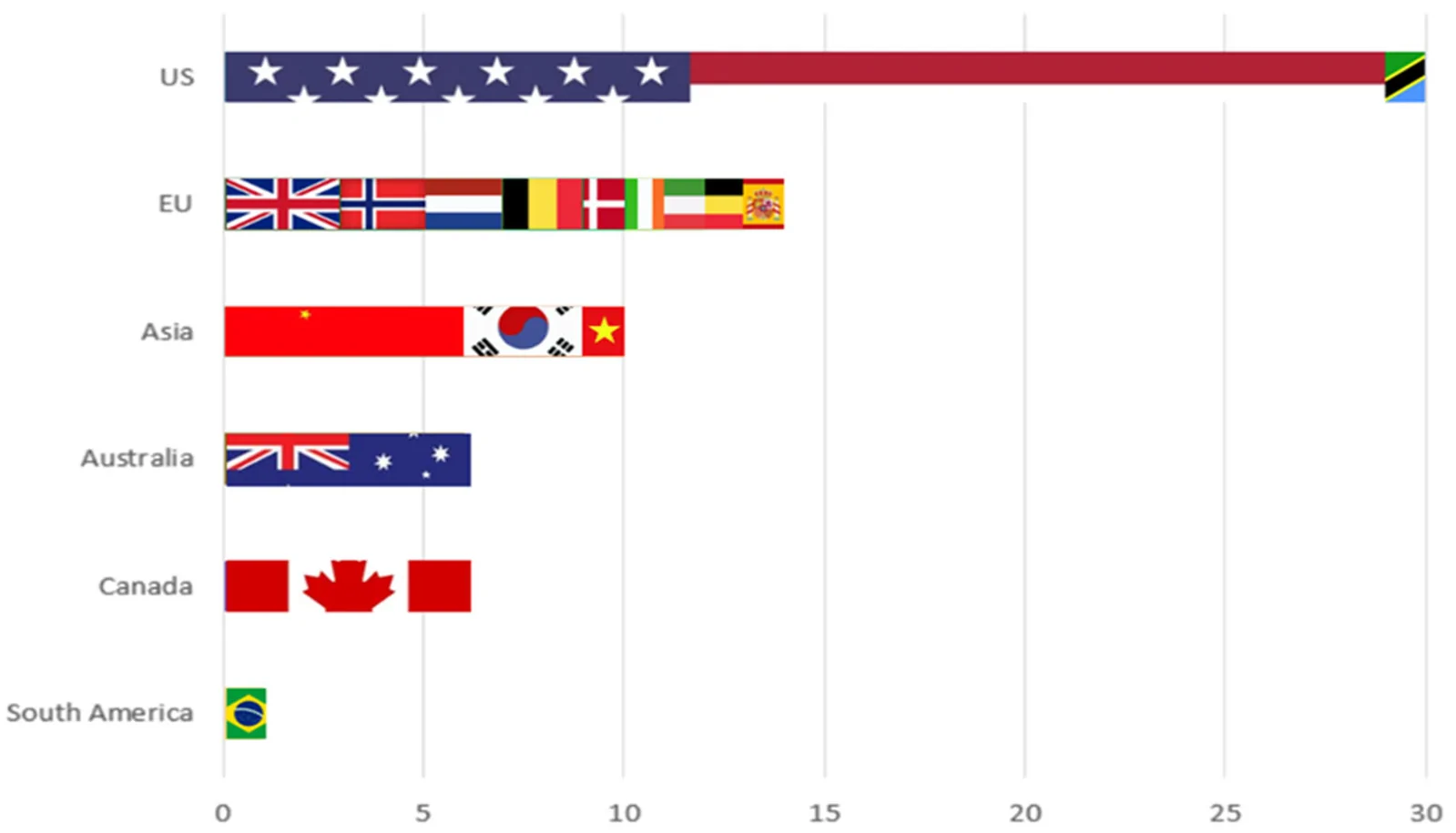
Distribution by country of affiliation. Countries are represented by flags and grouped as in Table S1. Tanzania is represented in the US bar, but separately, as the study has been conducted by an author with a primary affiliation in the US.

A total of 1784 articles were identified on PubMed. After removal of duplicates, 1733 titles and abstracts were screened. A total of 398 articles were eligible for full-text screening. A total of 323 articles were excluded during this step. Ten additional articles were identified through reference screening, out of which five were additionally included in this review. Data were extracted for 80 articles (Figure 1), of which 67 are either randomised clinical trials (n = 25, 37.3%), non-randomised trials, pilot or feasibility studies/trials, cohort studies, or other. Thirteen studies are meta-analyses (MA) or systematic reviews (SR) [37,38,39,40,41,42,43,44,45,46,47,48,49] (Supplementary File S3, Tables S1 and S2 for detailed reporting). In decreasing order, studies have been conducted in the following countries/regions: the United States (US) (n = 30, 44.78%) [50,51,52,53,54,55,56,57,58,59,60,61,62,63,64,65,66,67,68,69,70,71,72,73,74,75,76,77,78,79], Europe (n = 14, 20.9%) (Belgium, Denmark, Germany, Ireland, Italy, the Netherlands, Norway, Spain, UK) [80,81,82,83,84,85,86,87,88,89,90,91,92,93], Asia (n = 10, 14.93%) (China, Republic of Korea, Vietnam) [94,95,96,97,98,99,100,101,102,103], Australia (n = 6, 8.96%) [104,105,106,107,108,109], Canada (n = 6, 8.96%) [110,111,112,113,114,115], and South America (n = 1, 1.49%) (Brazil) [116]. Country classification was based on the first authors’ institutional affiliation, which in general was coherent with the country in which the study was conducted. However, using this approach, one study was classified under the United States [68], even though the study was conducted in Africa (Tanzania) (Figure 2).

### 3.1. Characteristics of Study Participants Among Included Studies (n = 67)

The largest proportion of interventions have been designed for breast cancer survivors (n = 26, 38.81%) [51,54,56,59,61,63,64,68,70,73,75,77,78,79,81,82,85,90,100,101,102,103,105,106,108,116]. Even those with mixed study populations (n = 27, 40.3%) often included a majority of breast cancer survivors [50,52,55,57,58,60,65,66,69,71,72,74,76,80,83,84,87,88,92,99,104,109,110,111,112,114,115] (e.g., 45.3% [55], 40% [115], 48% [88]). Other studies reporting results about one specific cancer type focused on colorectal cancer (including colon, rectum, anus) (n = 4, 5.97%) [53,67,94,95], gynaecological cancer (including cervix, ovarian and not specified subtypes) (n = 3, 4.48%) [89,91,93], lung cancer (n = 3, 4.48%) [96,97,98], prostate cancer (n = 2, 2.99%) [86,107], lymphoma (n = 1, 1.49%) [113], head and neck cancer (n = 1, 1.49%) [62]. The remaining studies included survivors from at least two different cancer types. Those who have not been previously cited are bladder, bone/soft tissue, brain, gastrointestinal, genitourinary, haematological/leukaemia, melanoma (including uveal melanoma), stomach and oesophageal, testicular, Merkel cell carcinoma, kidney, pancreatic, thyroid, and multiple myeloma. Across all included studies, a total of approximately 7880 participants were enrolled, with individual sample sizes ranging from 8 [74] to 587 [115]. Participants’ mean age ranged from 31.5 [113] to 70.7 [86] years. Regarding gender distribution, study participants’ gender has been reported, or could be reasonably assumed, based on the disease type (e.g., breast cancer survivors only). Overall, 6128 (77.7%) participants were female, 1751 (22.19%) were male, and gender was missing for one participant. About one third (n = 21, 31.34%) of interventions were fully remote e-health programmes or included telemedicine and technology-based or online elements (e.g., [51,54,56,62,81,82,83,86,87,89,90,102,106,107,108]).

### 3.2. Intervention Type and Modality

**Figure 3:**
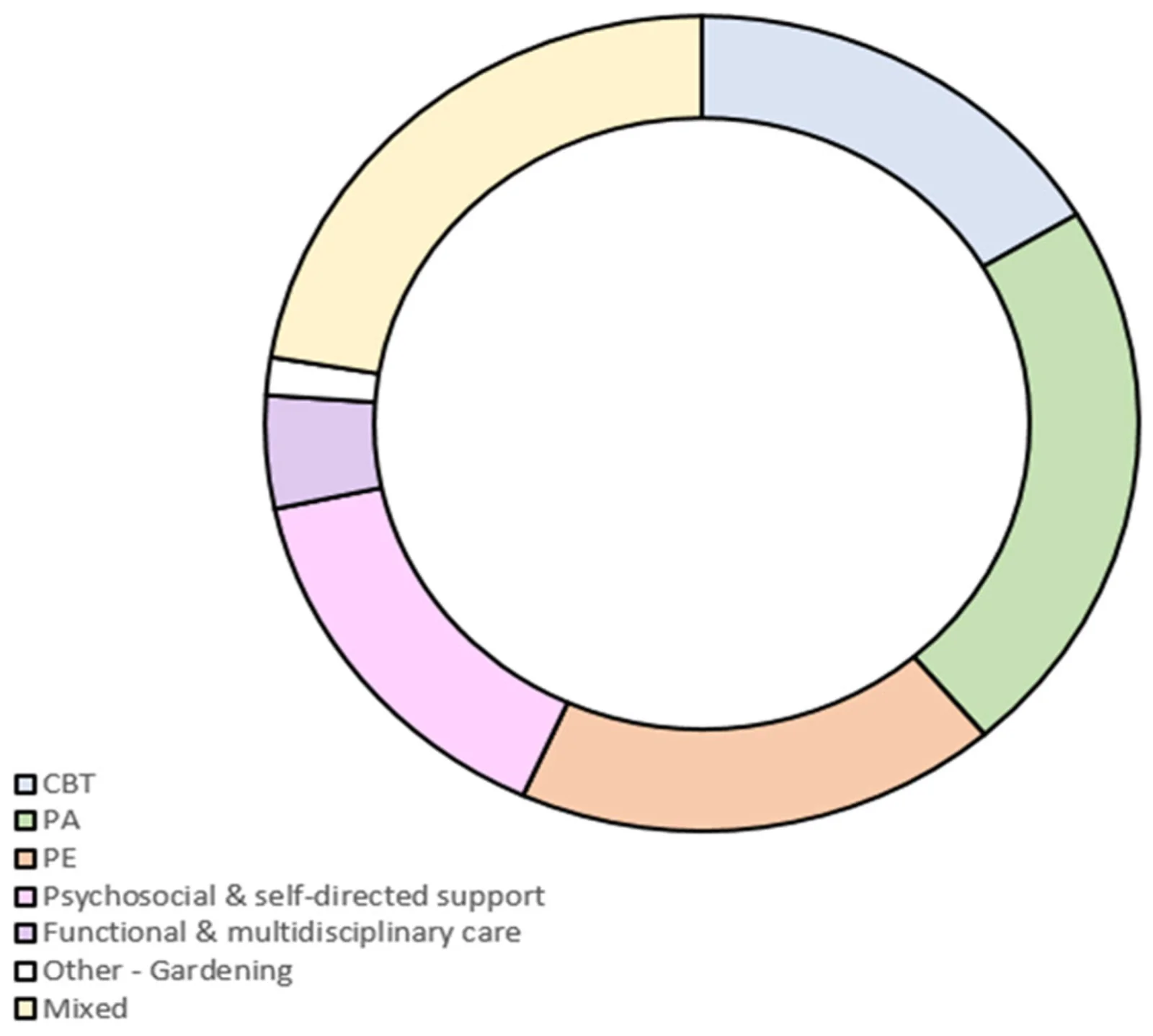
Distribution by type of evaluation.

Interventions commonly focused on one type of ‘activity’ such as PA (n = 15, 22.39%) [56,57,58,59,60,83,87,92,93,98,103,104,105,113,116], PE (n = 12, 17.91%) [61,62,63,64,65,66,67,68,69,101,106,107], various components of cognitive behavioural therapy (CBT) (n = 11, 16.42%) [50,51,52,53,54,55,88,91,108,109,110], psychosocial and self-directed support (n = 10, 14.93%) including art therapy (e.g., music therapy [97], a photography portrait intervention [85], an expressive writing intervention [100]) (n = 3, 4.48%), emotion focused or psychological interventions (n = 3, 4.48%) [81,82,96], self-management (n = 2, 2.99%) [84,102] and peer support (n = 2, 2.99%) [70,99]. Functional and multidisciplinary care interventions (n = 3, 4.48%) [71,89,112] and a single gardening intervention [72] have also been identified. The remaining studies (n = 15, 22.39%) combined several intervention components [73,74,75,76,77,78,79,80,86,90,94,95,111,114,115] (Figure 3).

Innovative examples of PA included a traditional Hawaiian dance (HULA) [57], Qigong [76], Yoga [51,74], mat Pilates [116], YMCA pickleball [60], Tai Chi [98,115], a city-wide walking path for cancer survivors [92]. Other methods included ‘Emotional Freedom Techniques’ [80], consisting of methods based on CBT, neurolinguistic programming and acupressure. One study investigated the effectiveness of traditional Chinese medicine in combination with group psychotherapy [95]. Another study evaluated a gardening experience where survivors would be matched with professional gardeners in order to develop a gardening activity at home [72]. Finally, a study investigated multidisciplinary integrative interventions in the context of an onco-sexual health clinic [112].

### 3.3. Measures

Most measures used in these studies are psychometrically validated instruments. Additionally, specific questionnaires have been added (for example, regarding satisfaction or usability), especially in pilot and feasibility studies. Some studies reported biological analyses, including cortisol [97], stool [57,95], saliva (e.g., [57,97]) and DNA [95]). Additionally, some studies included other biophysiological measures such as sleeping time, heart rate, and step count, mainly assessed by wearable devices, such as actigraphs or smartwatches [56,87,93,106].

### 3.4. Reported Psychosocial Outcomes

Study design, sample size, comparator, and outcome measures from the included studies were heterogeneous. Therefore, the present findings should be considered as a descriptive summary based on individual study results, rather than a combined analysis of effectiveness. Of the 67 included primary studies (systematic reviews and meta-analyses excluded from this count), n = 44 (65.67%) reported improvements in at least one psychosocial outcome, such as anxiety, depression, fatigue, distress, or FCR [50,51,53,54,58,59,60,62,63,64,65,66,68,69,71,72,73,74,75,78,80,81,82,84,85,92,93,95,96,97,98,99,100,101,102,104,105,107,108,109,111,112,115,116]. 18 studies (26.87%) reported mixed results, with improvements in some outcomes but not in all of them, or with comparable changes between intervention and control groups [52,55,56,57,61,67,76,77,79,83,87,88,89,94,103,110,113,114]. Some reported improvements did not remain stable over time (e.g., a decrease in FCR at two months post-intervention that did not fully persist at five months post-intervention [55]). Four studies reported no significant improvements in participants’ QoL [86,90,91,106]. One additional study reported poorer FCR, emotional well-being, energy and role functioning among participants with prolonged intervention use [70]. One breathing exercise intervention improved QoL (e.g., anxiety and depression symptoms) but participants also reported some minor discomforts such as dizziness, tiredness and yawning [108]. Because of the variety of measures used, these proportions should not be interpreted as a success rate for psychosocial interventions. Studies were classified according to the direction of effects reported by their authors; no independent assessment of statistical or clinical significance was performed.

### 3.5. Data Extracted from Systematic Reviews and Meta-Analysis

Regarding systematic reviews and meta-analyses, 13 reviews were included, of which six (46%) were systematic reviews [37,38,39,40,41,43], six were meta-analyses [42,44,45,46,47,48], and one (8%) was an integrative review [49]. Most studies exclusively included RCTs or other clinical trials [41,42,43,45,46,47,48]. Four combined RCTs with quasi-experimental or randomised studies [37,39,40,44], two broadened eligibility respectively to pilot and single-arm trials [38] or observational and qualitative studies [49] (Table S2). Most of them focused either on one intervention method (e.g., acceptance and commitment therapy (ACT) [38], yoga [42], or resistance training [47]) or one patient group (e.g., breast cancer survivors [39], head and neck cancer [40], or colorectal cancer [45]). The number of studies included ranged from 6 [41] to 132 [46], with a median value of 19 studies included. Most reviews (n = 8, 61.54%) reported positive psychosocial outcomes, including reduced fatigue, depression, anxiety, distress, and overall improved QoL. These outcomes were achieved through ACT [38], CBT [46], yoga [41], resistance training [47], and mindfulness-based interventions [48]. Three (23%) reported mixed or partial effects [39,40,42] with positive effects in some interventions/settings, but not in all. Another MA reported small but beneficial effects amongst an immigrant population [44]. Finally, an MA of PA interventions in colorectal cancer survivors reported no significant impact on health-related quality of life (HrQoL) outcomes [45].

## 4. Discussion

Previous reviews focusing on interventions for cancer survivors have often focused on a single intervention modality (e.g., CBT [39,43,46], ACT [38], yoga [42]) or on only one cancer type (e.g., breast [39] or head and neck cancer [40]). Currently existing reviews that have reported results on different intervention types or on various cancer diagnoses were conducted several years earlier. Additionally, they focused mainly on RCTs, which reduced the number of included studies [44,45,49]. The combination of 67 studies and 13 SRs or MAs, including various intervention types, published until 2025 offers a broader and more up-to-date overview of the rapidly evolving field of cancer care. Less common intervention types or male and rare cancer populations may gain more visibility through an approach that does not include RCTs exclusively. Through its broader reporting, this rapid ScR can help identify topics for future MAs on either specific interventions or groups of survivors. In this review, PA (22.39%), PE (17.9%), and cognitive behavioural therapy (16.4%) were the most common intervention types. This may be related to their endorsement in official guidelines, which may be linked to their precise methodology, evaluation through validated outcome measures, and their reproducibility (e.g., in trials) [117]. Regarding physical activity, its place in cancer survivorship care has recently been confirmed by trial results showing effects on disease outcomes (e.g., survival) in some cancer types [118].

Various consequences (e.g., physical, emotional, existential) from cancer and its treatments can persist for years after the end of active treatment. In consequence, they are likely to have a significant influence on cancer survivors’ psychosocial well-being and overall QoL. In fact, cancer survivors have lower levels of physical role and/or emotional functioning [119]. QoL remains altered five years after the diagnosis in around 45% of French cancer survivors. Gender and cancer diagnosis influence the exact percentage among different survivor groups [120].

In recent years, the consideration of co-occurring and interdependent symptoms enhances our understanding of cancer treatment and its consequences [121]. Interventions are frequently tailored to improve one specific outcome variable (e.g., improve PA, cognitive functioning). However, other psychosocial outcomes (e.g., social relations or other specific survivorship concerns) may also be improved by these interventions. Therefore, the future of psychosocial survivorship care requires further development, research, and implementation of multimodal interventions [121]. A stepped care approach may foster equal access to interventions, based on every survivor’s individual psychosocial needs. Survivors with higher distress may require blended or in-person interventions which could be delivered by specialised health-care professionals.

Furthermore, additional work may be needed in order to make sure these interventions are equally available and sustainable. Cost and effectiveness analyses are required in this context [121]. Regarding survivors’ participation in the interventions, the studies included in this review predominantly involved breast cancer survivors, while women represented roughly 78% of the overall study participants. This may be partially explained by gender roles and social constructions of masculinity, which can discourage emotional expression and contribute to feelings of embarrassment [122]. In fact, “men-only” or online interventions may sometimes provide higher perceived confidentiality and could therefore enhance adherence and trust, especially when it comes to sensitive topics [123]. Additionally, the literature suggests that future development and implementation should be particularly attentive to socioeconomically vulnerable and elderly survivors, as they often have various comorbidities, fewer resources, and less access to survivorship care [121].

However, geographic and socioeconomic inequalities continue to hinder access to psychosocial care [124]. Geographically isolated areas may have limited transportation modalities, with fewer specialised providers and financial toxicity further limiting access to supportive care [125]. Telehealth and online interventions may improve access to survivorship care [124], but they can also increase inequalities in individuals with limited digital literacy or unreliable internet access in some rural areas [126]. Hybrid or low-technology alternatives, like community-based programmes, may be worth considering alongside digital interventions [127]. More precisely, many of today’s survivorship interventions are developed in high-income countries, consistent with the results of the present ScR in which nearly half of the identified interventions have been developed in the United States (44.78%). Prior research has highlighted that QoL is correlated with gender, concurrent health issues, and education status [119]. As a consequence, development and implementation research should be a priority for these populations [128].

### Limits and Strengths

This rapid ScR has several limitations. Screening was limited to a single database (PubMed/MEDLINE). This methodology, although consistent with recommendations for a rapid review, may have limited the sensitivity of the search. Databases such as PsycINFO or Embase could be considered in a more exhaustive review. No formal risk-of-bias assessment was conducted, as the review’s objective was to map out existing literature rather than to analyse intervention effects. This is also consistent with published recommendations [129]. Furthermore, given that 6 original articles were also reported in the cited systematic reviews (SR) or meta-analyses (MA), some overlap in evidence cannot be excluded. Reporting of data extracted from SR and MA has therefore been provided separately from the remaining included studies. The inclusion of studies using a variety of measures remains interesting in the context of a ScR; however, for future MAs this should be considered. In fact, the inclusion of a wide range of intervention types and measures (including those with very small samples and marginal interventions) introduces an important heterogeneity in results. Therefore, no final conclusion on effect size and efficacy can be drawn. However, this methodology remains valuable for a broad mapping of interventions. Finally, eligibility was restricted to studies published in English, French, or German. This may have led to selection bias. Nevertheless, this review provides a broad, cross-diagnosis overview of various survivorship interventions with an impact on psychosocial outcome variables, regardless of whether they are under development or tested in an RCT. The combined inclusion of original articles and SRs or MAs allowed reporting of both already consolidated and emerging evidence. Resolution of disagreements through consensus discussions supported the rigour of the review process.

## 5. Conclusions

Most interventions have been developed for breast cancer patients or have included a majority of female patients (even interventions that have not been specifically designed for breast cancer patients). This is related to the high global prevalence of breast cancer as one of the main female cancer diagnoses. However, it seems important to enhance understanding regarding psychosocial needs and interventions in male cancer patients. Furthermore, developing interventions with a holistic approach using biophysiopsychological evaluation criteria seems crucial. The growing knowledge of symptom clusters and co-occurring symptoms supports the interest in multimodal interventions. Hence, interventions focusing on one symptom could potentially be beneficial to target specific symptoms. Finally, rigorous development, implementation, and cultural adaptations of e-health interventions seem crucial with regard to socioeconomic inequalities in accessing supportive cancer care. Eventually, addressing these gaps is not only a matter of improving cancer survivors’ QoL, but also of enhancing health equity and social justice.

## Data Availability

No new data were created or analysed in this study. Data sharing is not applicable to this article.

## Supplementary Materials

The following supporting information can be downloaded at: https://www.mdpi.com/article/10.3390/cancers18203385/s1, Supplementary File S1: PRISMA-ScR checklist; Supplementary File S2: Search strategy and documentation PubMed (MEDLINE); Supplementary File S3: Table S1: Summary of study results 2020–2025; Table S2: Summary of results extracted from systematic literature reviews and meta-analysis.

## Abbreviations

The following abbreviations are used in the manuscript

WHO	World Health Organization
QoL	Quality of life
APOS	American Society of Psycho-Oncology
PE	Psychoeducation
PA	Physical activity
CBT	Cognitive behavioural therapy
MA	Meta-analysis
SR	Systematic review
ScR	Scoping review
US	United States
EU	Europe
FCR	Fear of cancer recurrence
ACT	Acceptance and commitment therapy
RCT	Randomised controlled trial
HrQoL	Health-related quality of life

## References

[1] Global Cancer Burden Growing, Amidst Mounting Need for Services. https://www.who.int/news/item/01-02-2024-global-cancer-burden-growing--amidst-mounting-need-for-services.

[2] Vaz-Luis I., Masiero M., Cavaletti G., Cervantes A., Chlebowski R.T., Curigliano G., Felip E., Ferreira A.R., Ganz P.A., Hegarty J. (2022). ESMO Expert Consensus Statements on Cancer Survivorship: Promoting High-Quality Survivorship Care and Research in Europe. Ann. Oncol..

[3] Razavi D., Delvaux N., Bredart A., Autier P., Bron D., Debusscher L., Stryckmans P. (1993). Professional Rehabilitation of Lymphoma Patients: A Study of Psychosocial Factors Associated with Return to Work. Support. Care Cancer.

[4] Mullan F. (1985). Seasons of Survival: Reflections of a Physician with Cancer. N. Engl. J. Med..

[5] Miller K., Merry B., Miller J. (2008). Seasons of Survivorship Revisited. Cancer J..

[6] Mollica M.A., Doose M., Reed C., Tonorezos E. (2025). Defining Concepts in Cancer Survivorship. Cancer.

[7] Van Leeuwen M., Husson O., Alberti P., Arraras J.I., Chinot O.L., Costantini A., Darlington A.-S., Dirven L., Eichler M., On behalf of the EORTC QLG (2018). Understanding the Quality of Life (QOL) Issues in Survivors of Cancer: Towards the Development of an EORTC QOL Cancer Survivorship Questionnaire. Health Qual. Life Outcomes.

[8] Berry L.L., Davis S.W., Godfrey Flynn A., Landercasper J., Deming K.A. (2019). Is It Time to Reconsider the Term “Cancer Survivor”?. J. Psychosoc. Oncol..

[9] Colón Y. (2017). Survivorship: How to Survive Cancer and Still Lose Your Life. Clin. J. Oncol. Nurs..

[10] Kelly K.M., Shah N., Shedlosky-Shoemaker R., Porter K., Agnese D. (2011). Living Post Treatment: Definitions of Those with History and No History of Cancer. J. Cancer Surviv. Res. Pract..

[11] Feuerstein M. (2009). Work and Cancer Survivors.

[12] Le G.H., Hermansen Å., Dahl E. (2025). Return to Work after Cancer–the Impact of Working Conditions: A Norwegian Register-Based Study. J. Cancer Surviv..

[13] Andreu Y., Picazo C., Murgui S., Soto-Rubio A., García-Conde A., Romero R. (2023). Exploring the Independent Association of Employment Status to Cancer Survivors’ Health-Related Quality of Life. Health Qual. Life Outcomes.

[14] Wu W., Yackel H.D., Salner A., Chen M.-H., Judge M.P., Cong X., Xu W. (2024). Work-Health Balance of Cancer Survivors Returning to Work: A Meta-Ethnography. Eur. J. Oncol. Nurs..

[15] Jones J.M., Howell D., Longo C., Olson K., Bedard P., Amir E., Zheng S., Chow B., Avery L. (2025). The Association of Cancer-Related Fatigue on the Social, Vocational and Healthcare-Related Dimensions of Cancer Survivorship. J. Cancer Surviv..

[16] Yao G., Lai J.-S., Garcia S.F., Yount S., Cella D. (2023). Positive and Negative Psychosocial Impacts on Cancer Survivors. Sci. Rep..

[17] Faccio F., Renzi C., Giudice A.V., Pravettoni G. (2018). Family Resilience in the Oncology Setting: Development of an Integrative Framework. Front. Psychol..

[18] Acquati C., Miller-Sonet E., Zhang A., Ionescu E. (2023). Social Wellbeing in Cancer Survivorship: A Cross-Sectional Analysis of Self-Reported Relationship Closeness and Ambivalence from a Community Sample. Curr. Oncol..

[19] García-Vivar C., Rodriguez-Matesanz I. (2024). Long-Term Cancer Survivorship: A Family Affair. Cancer Care Res. Online.

[20] Howard-Jones G., Vassilev I., Fenlon D., Ewings S., Richardson A. (2022). Influence of Social Networks on Cancer Survivors’ Self-Management Support: A Mixed Methods Study. Eur. J. Cancer Care.

[21] Vrontaras N. (2018). Cancer Patients’ Views on the Family Changes and the Family Social Support. J. Eur. Psychol. Stud..

[22] Kim Y., Carver C.S., Ting A. (2019). Family Caregivers’ Unmet Needs in Long-Term Cancer Survivorship. Semin. Oncol. Nurs..

[23] Kornblith A.B., Ligibel J. (2003). Psychosocial and Sexual Functioning of Survivors of Breast Cancer. Semin. Oncol..

[24] Straker N. (2019). Psychodynamic Psychiatry for Patients with Cancer: Survivorship. Psychodyn. Psychiatry.

[25] Luigjes-Huizer Y.L., Tauber N.M., Humphris G., Kasparian N.A., Lam W.W.T., Lebel S., Simard S., Smith A.B., Zachariae R., Afiyanti Y. (2022). What Is the Prevalence of Fear of Cancer Recurrence in Cancer Survivors and Patients? A Systematic Review and Individual Participant Data Meta-analysis. Psychooncology.

[26] Kim H.-J., McGuire D.B., Tulman L., Barsevick A.M. (2005). Symptom Clusters: Concept Analysis and Clinical Implications for Cancer Nursing. Cancer Nurs..

[27] Falisi A.L., Wiseman K.P., Gaysynsky A., Scheideler J.K., Ramin D.A., Chou W.-Y.S. (2017). Social Media for Breast Cancer Survivors: A Literature Review. J. Cancer Surviv. Res. Pract..

[28] Zeng Y., Zhang J.-E., Cheng A.S.K., Cheng H., Wefel J.S. (2019). Meta-Analysis of the Efficacy of Virtual Reality-Based Interventions in Cancer-Related Symptom Management. Integr. Cancer Ther..

[29] Hernandez Silva E., Lawler S., Langbecker D. (2019). The Effectiveness of mHealth for Self-Management in Improving Pain, Psychological Distress, Fatigue, and Sleep in Cancer Survivors: A Systematic Review. J. Cancer Surviv. Res. Pract..

[30] Munn Z., Peters M.D.J., Stern C., Tufanaru C., McArthur A., Aromataris E. (2018). Systematic Review or Scoping Review? Guidance for Authors When Choosing between a Systematic or Scoping Review Approach. BMC Med. Res. Methodol..

[31] Levac D., Colquhoun H., O’Brien K.K. (2010). Scoping Studies: Advancing the Methodology. Implement. Sci..

[32] Hamel C., Michaud A., Thuku M., Skidmore B., Stevens A., Nussbaumer-Streit B., Garritty C. (2021). Defining Rapid Reviews: A Systematic Scoping Review and Thematic Analysis of Definitions and Defining Characteristics of Rapid Reviews. J. Clin. Epidemiol..

[33] Garritty C., Gartlehner G., Nussbaumer-Streit B., King V.J., Hamel C., Kamel C., Affengruber L., Stevens A. (2021). Cochrane Rapid Reviews Methods Group Offers Evidence-Informed Guidance to Conduct Rapid Reviews. J. Clin. Epidemiol..

[34] Augustine J.M., Lucas C. (2026). Systematic, Narrative, Scoping or Rapid Reviews—When, Why and How to Choose the Best Option in Pharmacy Education. Curr. Pharm. Teach. Learn..

[35] Moher D., Shamseer L., Clarke M., Ghersi D., Liberati A., Petticrew M., Shekelle P., Stewart L.A., PRISMA-P Group (2015). Preferred Reporting Items for Systematic Review and Meta-Analysis Protocols (PRISMA-P) 2015 Statement. Syst. Rev..

[36] Tricco A.C., Lillie E., Zarin W., O’Brien K.K., Colquhoun H., Levac D., Moher D., Peters M.D.J., Horsley T., Weeks L. (2018). PRISMA Extension for Scoping Reviews (PRISMA-ScR): Checklist and Explanation. Ann. Intern. Med..

[37] Hwang N.-K., Jung Y.-J., Park J.-S. (2020). Information and Communications Technology-Based Telehealth Approach for Occupational Therapy Interventions for Cancer Survivors: A Systematic Review. Healthcare.

[38] Mathew A., Doorenbos A.Z., Jang M.K., Hershberger P.E. (2021). Acceptance and Commitment Therapy in Adult Cancer Survivors: A Systematic Review and Conceptual Model. J. Cancer Surviv. Res. Pract..

[39] Ratcliff C.G., Torres D., Tullos E.A., Geng Y., Lu Q. (2021). A Systematic Review of Behavioral Interventions for Rural Breast Cancer Survivors. J. Behav. Med..

[40] Margalit D.N., Salz T., Venchiarutti R., Milley K., McNamara M., Chima S., Wong J., Druce P., Nekhlyudov L. (2022). Interventions for Head and Neck Cancer Survivors: Systematic Review. Head Neck.

[41] Giridharan S., Soumian S., Ansari J. (2024). Yoga for Cancer Survivors (YOCAS): A Systematic Review of the YOCAS Program’s Impact on Physical and Psychological Well-Being. Cureus.

[42] Armer J.S., Lutgendorf S.K. (2020). The Impact of Yoga on Fatigue in Cancer Survivorship: A Meta-Analysis. JNCI Cancer Spectr..

[43] Addison S., Shirima D., Aboagye-Mensah E.B., Dunovan S.G., Pascal E.Y., Lustberg M.B., Arthur E.K., Nolan T.S. (2022). Effects of Tandem Cognitive Behavioral Therapy and Healthy Lifestyle Interventions on Health-Related Outcomes in Cancer Survivors: A Systematic Review. J. Cancer Surviv. Res. Pract..

[44] Lui F., Lewicka M., Bao G.C., Moyer A., Boyce L., Leng J. (2023). A Systematic Review and Meta-Analysis of Psychosocial Interventions for Immigrant and Limited English Proficient Cancer Patients. Psychooncology.

[45] Abdul Razak N., Azhar Z.I., Baharuddin I.H., Ismail Z., Mohd Azman Z.A., Abdul Manap S.A., Ramli N. (2024). Does Exercise Improve Health-Related Quality of Life of Colorectal Cancer Survivors? A Systematic Review and Meta-Analysis. Asian Pac. J. Cancer Prev. APJCP.

[46] Dils A.T., O’Keefe K., Dakka N., Azar M., Chen M., Zhang A. (2024). The Efficacy of Cognitive Behavioral Therapy for Mental Health and Quality of Life among Individuals Diagnosed with Cancer: A Systematic Review and Meta-Analysis. Cancer Med..

[47] Gray L., Sindall P., Pearson S.J. (2024). Does Resistance Training Ameliorate Cancer-Related Fatigue in Cancer Survivors? A Systematic Review with Meta-Analysis. Disabil. Rehabil..

[48] Telles R., Whitney B.M., Froelich S., Lutgendorf S.K. (2024). Mindfulness-Based Psychosocial Interventions and Psychological Wellbeing in Cancer Survivorship: A Meta-Analysis. Health Psychol. Rev..

[49] Coyne E., Dieperink K.B., Voltelen B., da Silva Barreto M., Garcia-Vivar C. (2024). Posttreatment Health Interventions for Adult Cancer Survivors and Their Families: An Integrated Review. Support. Care Cancer.

[50] Victorson D., Murphy K., Benedict C., Horowitz B., Maletich C., Cordero E., Salsman J.M., Smith K., Sanford S. (2020). A Randomized Pilot Study of Mindfulness-Based Stress Reduction in a Young Adult Cancer Sample: Feasibility, Acceptability, and Changes in Patient Reported Outcomes. Psychooncology.

[51] Flanagan J.M., Post K., Hill R., Winters L.N. (2021). Findings from a Provider-Led, Mindfulness-Based, Internet-Streamed Yoga Video Addressing the Psychological Outcomes of Breast Cancer Survivors. Holist. Nurs. Pract..

[52] Arch J.J., Mitchell J.L., Genung S.R., Judd C.M., Andorsky D.J., Bricker J.B., Stanton A.L. (2021). Randomized Trial of Acceptance and Commitment Therapy for Anxious Cancer Survivors in Community Clinics: Outcomes and Moderators. J. Consult. Clin. Psychol..

[53] Kelleher S.A., Fisher H.M., Winger J.G., Somers T.J., Uronis H.E., Wright A.N., Keefe F.J. (2021). Feasibility, Engagement, and Acceptability of a Behavioral Pain Management Intervention for Colorectal Cancer Survivors with Pain and Psychological Distress: Data from a Pilot Randomized Controlled Trial. Support. Care Cancer.

[54] Henneghan A.M., Becker H., Phillips C., Kesler S. (2021). Sustained Effects of Mantra Meditation Compared to Music Listening on Neurocognitive Outcomes of Breast Cancer Survivors: A Brief Report of a Randomized Control Trial. J. Psychosom. Res..

[55] Hall D.L., Yeh G.Y., O’Cleirigh C., Wagner L.I., Peppercorn J., Denninger J., Hirschberg A.M., Lee H., Markwart M., Siefring E. (2024). Pilot Randomized Controlled Trial of IN FOCUS: A Mind-Body Resiliency Intervention for Fear of Cancer Recurrence. Psychooncology.

[56] Solk P., Song J., Welch W.A., Spring B., Cella D., Penedo F., Ackermann R., Courneya K.S., Siddique J., Freeman H. (2023). Effect of the Fit2Thrive Intervention on Patient-Reported Outcomes in Breast Cancer Survivors: A Randomized Full Factorial Trial. Ann. Behav. Med. Publ. Soc. Behav. Med..

[57] Bantum E.O., Yamada P.M., Makolo T., Yu H., Pagano I., Subia N., Walsh C., Loo L.W.M. (2023). Hula as a Physical Activity and Social Support Intervention for Sustained Activity in Female Breast and Gynecologic Cancer Survivors. Front. Psychol..

[58] Beebe-Dimmer J.L., Finlay D.G., Ruterbusch J.J., Baird T., Simon M.S., Abrams J., Harper F.W.K., Podgorski I., Heath E.I. (2023). The Impact of High Intensity Interval Training in a Diverse Group of Cancer Survivors: CAPABLE, a Pilot Study. Prev. Med. Rep..

[59] Lee C.Y., Laffoon K., Mama S.K., Liao Y., Whisenant M., Brewster A., Mendoza T., Mitchell S.J., Tracy P.V., Garza G.M. (2024). Outcomes for Breast Cancer Survivors with Metastatic Disease in a Physical Activity Program for Medically Underserved Cancer Survivors. J. Cancer Surviv. Res. Pract..

[60] Parker N.H., de Cerqueira Santos A., Mintrone R., Turner K., Sutton S.K., O’Connor T., Huang J., Lael M., Cruff S., Grassia K. (2025). Feasibility, Acceptability, and Outcomes of Project Rally: Pilot Study of a YMCA-Based Pickleball Program for Cancer Survivors. Healthcare.

[61] Im E.-O., Kim S., Yang Y.L., Chee W. (2020). The Efficacy of a Technology-Based Information and Coaching/Support Program on Pain and Symptoms in Asian American Survivors of Breast Cancer. Cancer.

[62] Manne S., Hudson S., Frederick S., Mitarotondo A., Baredes S., Kalyoussef E., Ohman-Strickland P., Kashy D.A. (2020). E-Health Self-Management Intervention for Oral and Oropharyngeal Cancer Survivors: Design and Single-Arm Pilot Study of Empowered Survivor. Head Neck.

[63] Ashing K.T., George M. (2020). Exploring the Efficacy of a Paraprofessional Delivered Telephonic Psychoeducational Intervention on Emotional Well-Being in African American Breast Cancer Survivors. Support. Care Cancer.

[64] Im E.-O., Yi J.-S., Kim H., Chee W. (2021). A Technology-Based Information and Coaching/Support Program and Self-Efficacy of Asian American Breast Cancer Survivors. Res. Nurs. Health.

[65] Syrjala K.L., Walsh C.A., Yi J.C., Leisenring W.M., Rajotte E.J., Voutsinas J., Ganz P.A., Jacobs L.A., Palmer S.C., Partridge A. (2022). Cancer Survivorship Care for Young Adults: A Risk-Stratified, Multicenter Randomized Controlled Trial to Improve Symptoms. J. Cancer Surviv. Res. Pract..

[66] Sohl S.J., Sadasivam R.S., Kittel C., Dressler E.V., Wentworth S., Balakrishnan K., Weaver K.E., Dellinger R.A., Puccinelli-Ortega N., Cutrona S.L. (2023). Pilot Study of Implementing the Shared Healthcare Actions & Reflections Electronic Systems in Survivorship (SHARE-S) Program in Coordination with Clinical Care. Cancer Med..

[67] Wang T.-J., Chang S.-C., Hsu H.-H., Huang C.-S., Lin T.-R., Lin Y.-P., Chang K.-S. (2023). Efficacy of a Self-Management Program on Quality of Life in Colorectal Cancer Patients: A Randomized Controlled Trial. Eur. J. Oncol. Nurs..

[68] Gutnik L., Msoka E.F., Dwarampudi S., Hollis T., McLeod M.C., Locke J.E., Scarinci I., Rocque G.B., Mremi A., Serventi F. (2024). Meaningful Engagement of People Living with Cancer: Leveraging Breast Cancer Survivors in a Stigma Reduction Intervention in Tanzania. World J. Surg..

[69] Berzins N.J., Mackenzie M., Galantino M.L., Pickles N., Hebbel S., Leonard T., Beneck D., Peterson M. (2024). Preliminary Effectiveness Study of a Community-Based Wellness Coaching for Cancer Survivors Program. Am. J. Lifestyle Med..

[70] Thompson T., Pérez M., Yan Y., Kreuter M.W., Margenthaler J.A., Colditz G.A., Jeffe D.B. (1982). Randomized Controlled Trial of a Breast Cancer Survivor Stories Intervention for African American Women. Soc. Sci. Med..

[71] Polo K.M., Moore E.S., Sommers S.H. (2022). Exploring the Impact of the Occupational Therapy Health and Wellness Program (OT-HAWP) on Performance and the Health-Related Quality of Life of Cancer Survivors. Occup. Ther. Health Care.

[72] Blair C.K., Adsul P., Guest D.D., Sussman A.L., Cook L.S., Harding E.M., Rodman J., Duff D., Burgess E., Quezada K. (2021). Southwest Harvest for Health: An Adapted Mentored Vegetable Gardening Intervention for Cancer Survivors. Nutrients.

[73] Dhruva A., Wu C., Miaskowski C., Hartogensis W., Rugo H.S., Adler S.R., Kaptchuk T.J., Kelkar R., Agarawal S., Vadodaria A. (2020). A 4-Month Whole-Systems Ayurvedic Medicine Nutrition and Lifestyle Intervention Is Feasible and Acceptable for Breast Cancer Survivors: Results of a Single-Arm Pilot Clinical Trial. Glob. Adv. Health Med..

[74] Galantino M.L., Brooks J., Tiger R., Jang S., Wilson K. (2020). Effectiveness of Somatic Yoga and Meditation: A Pilot Study in a Multicultural Cancer Survivor Population with Chemotherapy-Induced Peripheral Neuropathy. Int. J. Yoga Ther..

[75] Tami-Maury I.M., Liao Y., Rangel M.L., Gatus L.A., Shinn E.H., Alexander A., Basen-Engquist K. (2022). Active Living After Cancer: Adaptation and Evaluation of a Community-Based Physical Activity Program for Minority and Medically Underserved Breast Cancer Survivors. Cancer.

[76] Zimmerman C.S., Temereanca S., Daniels D., Penner C., Cannonier T., Jones S.R., Kerr C. (2023). A Randomized Controlled Pilot Trial Comparing Effects of Qigong and Exercise/Nutrition Training on Fatigue and Other Outcomes in Female Cancer Survivors. Integr. Cancer Ther..

[77] Allison K.C., McCuen-Wurst C., Raevsky A., Holmes N., Goldbach M., Guerra C.E., Rendle K.A., Cadet T.J., Krouse R.S., Tchou J. (2024). The Group-based Telehealth Behavioral Weight Loss Program Among Breast Cancer Survivors: A Pilot and Feasibility Study. Obes. Sci. Pract..

[78] Crespi C.M., Ganz P.A., Partridge A.H., Wolff A., Joffe H., Irwin M.R., Thure K., Petersen L., Shih Y.-C.T., Bower J.E. (2024). Work Productivity Among Younger Breast Cancer Survivors: The Impact of Behavioral Interventions for Depression. Value Health J. Int. Soc. Pharmacoeconomics Outcomes Res..

[79] Tyrus Korecki J.R., Ganz P.A., Partridge A.H., Wolff A.C., Petersen L., Crespi C.M., Bower J.E. (2024). Moderators of Intervention Efficacy in the Pathways to Wellness Trial of Survivorship Education and Mindfulness Meditation for Younger Breast Cancer Survivors. JCO Oncol. Pract..

[80] Tack L., Lefebvre T., Lycke M., Langenaeken C., Fontaine C., Borms M., Hanssens M., Knops C., Meryck K., Boterberg T. (2021). A Randomised Wait-List Controlled Trial to Evaluate Emotional Freedom Techniques for Self-Reported Cancer-Related Cognitive Impairment in Cancer Survivors (EMOTICON). EClinicalMedicine.

[81] Merckaert I., Waroquier P., Caillier M., Verkaeren O., Righes S., Liénard A., Libert Y., Kristanto P., Razavi D. (2023). Improving Emotion Regulation in Breast Cancer Patients in the Early Survivorship Period: Efficacy of a Brief Ecologically Boosted Group Intervention. Psychooncology.

[82] Tauber N.M., O’Toole M.S., Jensen A.B., Butow P.N., Thewes B., Elkjaer E., Knutzen S., von Heymann A., Johansen C., Zachariae R. (2023). ConquerFear-Group: A Randomized Controlled Trial of an Online-Delivered Group-Based Psychological Intervention for Fear of Cancer Recurrence in Breast Cancer Survivors. Psychooncology.

[83] Krell V., Porst J., Hafermann L., Kuhn J., Greiß F., Römer C., Wolfarth B. (2024). Telemedicine-Based Exercise Intervention in Cancer Survivors: A Non-Randomized Controlled Trial. Sci. Rep..

[84] Boland L., Bennett K.E., Cuffe S., Grant C., Kennedy M.J., Connolly D. (2023). Feasibility Randomised Control Trial of OptiMal: A Self-Management Intervention for Cancer Survivors. Curr. Oncol..

[85] Vagnini D., Sorge A., Acquati C., Colafemmina F.A., Saita E. (2024). Implementing the Framed Portrait Experience with Italian Breast Cancer Survivors: A Pilot Study Assessing Short Term Effects of an Existential Approach to Body Image, Coping Skills, and Self-Efficacy. J. Cancer Surviv. Res. Pract..

[86] Langlais C.S., Chen Y.-H., Van Blarigan E.L., Kenfield S.A., Kessler E.R., Daniel K., Ramsdill J.W., Beer T.M., Graff R.E., Paich K. (2022). Quality of Life of Prostate Cancer Survivors Participating in a Remotely Delivered Web-Based Behavioral Intervention Pilot Randomized Trial. Integr. Cancer Ther..

[87] Golsteijn R.H.J., Bolman C., Peels D.A., Volders E., de Vries H., Lechner L. (2023). Long-Term Efficacy of a Computer-Tailored Physical Activity Intervention for Prostate and Colorectal Cancer Patients and Survivors: A Randomized Controlled Trial. J. Sport Health Sci..

[88] Børøsund E., Ehlers S.L., Varsi C., Clark M.M., Andrykowski M.A., Cvancarova M., Solberg Nes L. (2020). Results from a Randomized Controlled Trial Testing *StressProffen*; an Application-based Stress-management Intervention for Cancer Survivors. Cancer Med..

[89] Areklett E.W., Hagen B.I., Stubberud J., Fagereng E., Andersson S., Lindemann K. (2024). Digital Goal Management Training for Cognitive Impairment in Cervical Cancer Survivors-a Randomized Comparison Study. J. Cancer Surviv. Res. Pract..

[90] Medina J.C., Flix-Valle A., Rodríguez-Ortega A., Hernández-Ribas R., Lleras De Frutos M., Ochoa-Arnedo C. (2022). ICOnnecta’t: Development and Initial Results of a Stepped Psychosocial eHealth Ecosystem to Facilitate Risk Assessment and Prevention of Early Emotional Distress in Breast Cancer Survivors’ Journey. Cancers.

[91] Hulbert-Williams N.J., Hulbert-Williams L., Flynn R.J., Pendrous R., MacDonald-Smith C., Mullard A., Swash B., Evans G., Price A. (2021). Evaluating Process and Effectiveness of a Low-Intensity CBT Intervention for Women with Gynaecological Cancer (the EPELIT Trial). AMRC Open Res..

[92] Humphreys L., Frith G., Humphreys H., Crank H., Dixey J., Greenfield D.M., Reece L.J. (2023). Evaluation of a City-Wide Physical Activity Pathway for People Affected by Cancer: The Active Everyday Service. Support. Care Cancer.

[93] Millet N., McDermott H.J., Munir F., Edwardson C.L., Plekhanova T., Moss E.L. (2025). The Feasibility and Acceptability of Delivering and Evaluating a Physical Activity Intervention (ACCEPTANCE) for Cervical Cancer Survivors. Pilot Feasibility Stud..

[94] Ho M., Ho J.W.C., Fong D.Y.T., Lee C.F., Macfarlane D.J., Cerin E., Lee A.M., Leung S., Chan W.Y.Y., Leung I.P.F. (2020). Effects of Dietary and Physical Activity Interventions on Generic and Cancer-Specific Health-Related Quality of Life, Anxiety, and Depression in Colorectal Cancer Survivors: A Randomized Controlled Trial. J. Cancer Surviv. Res. Pract..

[95] Sun L., Pang Y., Wang Z., Liu J., Peng R., Yan Y., Yang Y., Tang L. (2023). Effect of Traditional Chinese Medicine Combined Group Psychotherapy on Psychological Distress Management and Gut Micro-Biome Regulation for Colorectal Cancer Survivors: A Single-Arm Phase I Clinical Trial. Support. Care Cancer.

[96] Zhao J., Wang M., Huang R., Xu J., Gan C., Yu S., Tang L., Yao S., Li W., Cheng H. (2023). Effects of CALM Intervention on Neutrophil-to-Lymphocyte Ratio (NLR), Fear of Cancer Recurrence and Quality of Life in Patients with Lung Cancer. Support. Care Cancer.

[97] Du Y.-J., Guan Y.-H., Thome K.T., Dong J.-C. (2024). Music Therapy and Music Intervention for NSCLC Patients Undergoing PET with Fear of Cancer Recurrence. Integr. Cancer Ther..

[98] Takemura N., Cheung D.S.T., Fong D.Y.T., Lee A.W.M., Lam T.-C., Ho J.C.-M., Kam T.Y., Chik J.Y.K., Lin C.-C. (2024). Effectiveness of Aerobic Exercise and Tai Chi Interventions on Sleep Quality in Patients with Advanced Lung Cancer: A Randomized Clinical Trial. JAMA Oncol..

[99] Le P.D., Taylor C., Do M.T., Monahan R., Lee S., Sigireddi M., Wang C., Cabanes A., Ginsburg O., Tran T.H.T. (2024). Evaluation of the Stronger Together Peer Mentoring Model Among Patients with Breast and Gynecologic Cancer in Viet Nam. JCO Glob. Oncol..

[100] Ji L.-L., Lu Q., Wang L.-J., Sun X.-L., Wang H.-D., Han B.-X., Ma Y.-F., Lu G.-H. (2020). The Benefits of Expressive Writing among Newly Diagnosed Mainland Chinese Breast Cancer Patients. J. Behav. Med..

[101] Cheon J., Choi Y., Kim J.S., Ko B.K., Kim C.R., Kim J.H., Koh S.-J. (2022). “Teachable Moment”: Effects of an Educational Program on Knowledge and Quality of Life of Korean Breast Cancer Survivors. J. Cancer Educ..

[102] Kim S.M., Kim D.S., Jang Y., Kim M.K., Yu E.-S., Han D.H., Kim H.J. (2025). Evaluating the Effectiveness of a Mobile App for Breast Cancer Self-Management on Self-Efficacy: Nonrandomized Intervention Trial. JMIR MHealth UHealth.

[103] Kim S., Ko Y.H., Song Y., Kang M.J., Lee H., Kim S.H., Jeon J.Y., Cho Y.U., Yi G., Han J. (2020). Pre-Post Analysis of a Social Capital-Based Exercise Adherence Intervention for Breast Cancer Survivors with Moderate Fatigue: A Randomized Controlled Trial. Support. Care Cancer.

[104] Rutherford Z., Zwolinsky S., Kime N., Pringle A. (2021). A Mixed-Methods Evaluation of CARE (Cancer and Rehabilitation Exercise): A Physical Activity and Health Intervention, Delivered in a Community Football Trust. Int. J. Environ. Res. Public. Health.

[105] Macdonald E.R., Clifford B.K., Simar D., Ward R.E. (2022). Ballet after Breast Cancer: Investigating the Feasibility and Acceptability of a Novel 16-Week Classical Ballet Intervention for Breast Cancer Survivors. Support. Care Cancer.

[106] Singleton A.C., Raeside R., Partridge S.R., Tat-Ko J., Che Mun Sum S., Hyun K.K., Hayes M., Chow C.K., Thiagalingam A., Maka K. (2022). Supporting Breast Cancer Survivors via Text Messages: Reach, Acceptability, and Utility of EMPOWER-SMS. J. Cancer Surviv. Res. Pract..

[107] Heneka N., Chambers S.K., Schaefer I., Carmont K., Parcell M., Wallis S., Walker S., Tuffaha H., Steele M., Dunn J. (2024). Acceptability of a Virtual Prostate Cancer Survivorship Care Model in Rural Australia: A Multi-Methods, Single-Centre Feasibility Pilot. Aust. J. Rural Health.

[108] Wang H., Kwok W.H., Yao L.-Q., Liu X.-L., Bressington D., Chen M.-L., Huang H.-Q., Wang T., Tan J.-Y.B. (2024). An Evidence-Based Breathing Exercise Intervention for Chronic Pain Management in Breast Cancer Survivors: A Phase II Randomized Controlled Trial. Eur. J. Oncol. Nurs..

[109] Tran M.J., Jefford M., Fua T., Smith B., McDowell L., Dhillon H.M., Lynch F., Shaw J., White A., Wiesenfeld D. (2025). Feasibility and Acceptability of the Fear-Less Screening and Stratified-Care Model for Fear of Cancer Recurrence Among People Affected by Early-Stage Cancer. Psychooncology.

[110] Dirkse D., Hadjistavropoulos H.D., Alberts N.A., Karin E., Schneider L.H., Titov N., Dear B.F. (2020). Making Internet-Delivered Cognitive Behaviour Therapy Scalable for Cancer Survivors: A Randomized Non-Inferiority Trial of Self-Guided and Technician-Guided Therapy. J. Cancer Surviv. Res. Pract..

[111] Jammu A., Chasen M., van Heest R., Hollingshead S., Kaushik D., Gill H., Bhargava R. (2020). Effects of a Cancer Survivorship Clinic-Preliminary Results. Support. Care Cancer.

[112] Walker L.M., Sears C.S., Booker R., Doll C., Glaze S., Phan T., Brennan K., Millman R.D., Robinson J.W. (2021). Development, Implementation, and Evaluation of a Multidisciplinary Oncology Sexual Health Clinic in a Canadian Cancer Care Setting. J. Cancer Surviv. Res. Pract..

[113] Angelillo C., Tock W.L., Salaciak M., Reid R.E.R., Andersen R.E., Maheu C., Johnson N.A. (2024). A Single-Armed Proof-of-Concept Study of Lymfit: A Personalized, Virtual Exercise Intervention to Improve Health Outcomes in Lymphoma Survivors in the Pandemic. PLoS ONE.

[114] Ilie G., Knapp G., Davidson A., Snow S., Dahn H.M., MacDonald C., Tsirigotis M., Rutledge R.D.H. (2024). The Cancer Patient Empowerment Program: A Comprehensive Approach to Reducing Psychological Distress in Cancer Survivors, with Insights from a Mixed-Model Analysis, Including Implications for Breast Cancer Patients. Cancers.

[115] Carlson L.E., Jones J.M., Oberoi D., Piedalue K.-A., Wayne P.M., Santa Mina D., Lawal O.A., Speca M. (2025). Mindfulness and Tai Chi for Cancer Health (MATCH) Study: Primary Outcomes of a Preference-Based Multisite Randomized Comparative Effectiveness Trial. J. Clin. Oncol..

[116] Bertoli J., Bandeira A.C.N., de la Freitas C.R., Freitas Júnior I.F. (2025). Can Mat Pilates Improve Quality of Life Components in Breast Cancer Survivors Receiving Hormone Therapy? A Randomized Controlled Trial. Complement. Ther. Clin. Pract..

[117] Andersen B.L., Lacchetti C., Ashing K., Berek J.S., Berman B.S., Bolte S., Dizon D.S., Given B., Nekhlyudov L., Pirl W. (2023). Management of Anxiety and Depression in Adult Survivors of Cancer: ASCO Guideline Update. J. Clin. Oncol..

[118] Courneya K.S., Vardy J.L., O’Callaghan C.J., Gill S., Friedenreich C.M., Wong R.K.S., Dhillon H.M., Coyle V., Chua N.S., Jonker D.J. (2025). Structured Exercise after Adjuvant Chemotherapy for Colon Cancer. N. Engl. J. Med..

[119] Annunziata M.A., Muzzatti B., Flaiban C., Gipponi K., Carnaghi C., Tralongo P., Caruso M., Cavina R., Tirelli U. (2018). Long-Term Quality of Life Profile in Oncology: A Comparison between Cancer Survivors and the General Population. Support. Care Cancer.

[120] (2018). INCA La vie Cinq ans Après un Diagnostic de Cancer. https://www.cancer-environnement.fr/app/uploads/2025/01/INCa-2018_VICAN-5_La-vie-5-ans-apres-un-diagnostic-de-cancer.pdf.

[121] Aaronson N.K., Mattioli V., Minton O., Weis J., Johansen C., Dalton S.O., Verdonck-de Leeuw I.M., Stein K.D., Alfano C.M., Mehnert A. (2014). Beyond Treatment—Psychosocial and Behavioural Issues in Cancer Survivorship Research and Practice. Eur. J. Cancer Suppl..

[122] Zafar A., Baessler F., Ihrig A., Mayer G., Bugaj T.J., Maatouk I., Staeudle J., Friederich H., Schultz J. (2022). Barriers to Access Cancer-related Services for Men in High-income Countries: A Narrative Review Looking beyond Socioeconomic Disadvantages. Aging Cancer.

[123] Schover L.R., Canada A.L., Yuan Y., Sui D., Neese L., Jenkins R., Rhodes M.M. (2012). A Randomized Trial of Internet-based versus Traditional Sexual Counseling for Couples after Localized Prostate Cancer Treatment. Cancer.

[124] Alford-Teaster J., Vaclavik D., Imset I., Schiffelbein J., Lyons K., Kapadia N., Olson A., McGrath E.B., Schifferdecker K., Onega T. (2025). From Active Treatment to Surveillance: How the Barriers and Facilitators of Implementing Survivorship Care Planning Could Be an Opportunity for Telehealth in Oncology Care for Rural Patients. J. Cancer Surviv. Res. Pract..

[125] Chan A., Ke Y., Tanay M., Dagsi M., Bergerot C., Dixit N., Eng L., Cardeña Gutiérrez A., Jiang C., Velázquez A.I. (2024). Financial Toxicity in Cancer Supportive Care: An International Survey. JCO Glob. Oncol..

[126] DeGuzman P.B., Bernacchi V., Cupp C.A., Dunn B., Ghamandi B.J.F., Hinton I.D., Jameson M.J., Lewandowski D.L., Sheffield C. (2020). Beyond Broadband: Digital Inclusion as a Driver of Inequities in Access to Rural Cancer Care. J. Cancer Surviv. Res. Pract..

[127] Baxter M.F., Newman R., Longpré S.M., Polo K.M. (2017). Occupational Therapy’s Role in Cancer Survivorship as a Chronic Condition. Am. J. Occup. Ther..

[128] Koech M.J., Mwangi J., Kithaka B., Kimaru S., Kusu N., Munyi L., Chahonyo S., Makokha F. (2024). Effects of Stigma on Quality of Life of Cancer Survivors: Preliminary Evidence from a Survivorship Programme in Kenya. Heliyon.

[129] Aromataris E., Lockwood C., Porritt K., Pilla B., Jordan Z. (2024). JBI Manual for Evidence Synthesis.

