# Supplementary material for: Psychosocial Interventions in Cancer Survivorship: A Rapid Scoping Review of the Recent PubMed-Indexed Literature
Source: Cancers (Basel). 2026 Oct 20;18(20):3385. doi: 10.3390/cancers18203385 (PMC13703252; doi:10.3390/cancers18203385)
Supplement: Supplementary file 1 [file cancers-18-03385-s001.zip › Supplementary file S2_Search strategy.pdf]

Supplementary material S2. Search strategy and documentation **PubMed (MEDLINE)**

Search nr	Term
Concept 1	
1	Cancer Survivors OR Cancer Survivorship [MeSH]
2	“cancer survivorship” OR "cancer survivor" OR "cancer survivors" OR "cancer surveillance" OR "après-cancer"[Title/Abstract]
3	1 OR 2
Concept 2	
4	quality of life OR anxiety OR depression [MeSH]
5	anxiety OR distress OR depression OR "depressive symptoms" OR "psychological well-being" OR "psychological wellbeing" OR sleep OR insomnia OR psychosocial difficult* OR "quality of life" OR psycho* OR fear of cancer recurrence OR FCR
6	4 OR 5
Concept 3	
7	Psychosocial Intervention [MeSH]
8	intervention* OR therap* OR ehealth OR "e-health" OR telehealth OR "complex intervention" OR "blended intervention" OR selfhelp OR "self-help" OR application*
9	7 OR 8
10	2020:2025 [dp]
Final search	3 AND 6 AND 9 AND 10

Search period: 2020-2025

Inclusion and exclusion criteria:

<table>
<tr><th>Inclusion</th><th>Exclusion</th></tr>
<tr><td>
Population: adult cancer survivors (≥18 years), regardless of cancer diagnosis.

Language: English, French, or German

Study designs: experimental studies, cohort studies, pilot studies, feasibility studies, systematic reviews (SRs), and meta-analyses (MAs).

Intervention: studies evaluating exposure to a defined psychosocial intervention, including:

psychoeducation (PE);
physical activity (PA);
cognitive behavioural therapy (CBT);
psychological interventions;
self-directed support interventions;
functional or multidisciplinary care;
other interventions including at least one of the above-mentioned psychosocial components

Psychosocial outcomes: studies had to report at least one sufficiently detailed psychosocial outcome.
</td><td>

Studies involving participants younger than 18 years.
Studies exclusively involving adult survivors of paediatric/childhood cancer.
Studies involving participants without a cancer diagnosis.
Observational cohort studies describing psychosocial outcomes without an intervention component.
Studies without clear or sufficiently detailed reporting of psychosocial outcomes.
Studies not evaluating a psychosocial intervention.
Umbrella reviews, case reports and case studies.
Publications outside the 2020–2025 period.
Publications in languages other than English, French, or German.

</td></tr>
</table>
