# Supplementary material for: Psychosocial Interventions in Cancer Survivorship: A Rapid Scoping Review of the Recent PubMed-Indexed Literature
Source: Cancers (Basel). 2026 Oct 20;18(20):3385. doi: 10.3390/cancers18203385 (PMC13703252; doi:10.3390/cancers18203385)
Supplement: Supplementary file 1 [file cancers-18-03385-s001.zip › Supplementary file S3_Table S1 and table S2.pdf]

Supplementary S3 Table S1 and Table S2

Table S1. Summary of study results 2020 – 2025.

Year	Author	Measures*	Participants	Age	Intervention	Intervention Type	Cancer	Psychosocial outcomes	Study Type	Trial Registration	Country
2020	Dirkse et al. [110]	GAD-7, PHQ-9, SBQ-R, FCRI-SF	14 m, 69 f	50.80 (13.17)	ICBT programme (Wellbeing After Cancer) self-guided versus technician guided	CBT	Gynaecological, Gastrointestinal, Breast, Haematological, Other not specified	Improvement of anxiety, depression, FCR in both groups	RCT (Randomised non-inferiority trial)	ISRCTN32477700	Canada
2020	Børøsund et al. [88]	PSS-14, HADS, SF-36-Item, RAND36 version	141 f, 31 m	52 (11.2)	StressProffen	CBT (eHealth stress management)	Breast (48%), Brain, Prostate, Lymphoma, Colon, Other, not reported	PSS-14: Improvements in intervention group. HADS: no differences between groups. SF-36: improved physical functioning, role functioning, motional functioning, social functioning and mental health in intervention group.	RCT	Not reported	Norway
2020	Victorson et al. [50]	PROMIS, Computer Adaptive Tests (CATs)	99 f, 27 m	IG: 33.01 (4.42), CG: 32.56 (5.14)	mindfulness-based stress reduction	CBT (mind-body)	Bone/soft tissue, Brain, Breast, Colorectal, Head and neck, Leukaemia, Lung, Lymphoma, Melanoma, Gynaecological, Stomach and oesophageal, Testicular, Other not specified	Intervention Group: HrQoL improvements (compared to Control Group (CG))	RCT	NCT02495376	US
2021	Flanagan et al. [51]	GAD, WEMWBS, KPCSF	35 f	56.17 (12.17)	Yoga video (internet streamed)	CBT (mind-body)	Breast	MWB: improved mental well-being; KPCSF: increased knowledge; GAD: reduced anxiety	Cohort Study (single-arm, pre versus post-intervention)	Not reported	US
2021	Arch et al. [52]	HADS-A, CARS, IES-R, CESD, FACIT, RAND SF-36	118 f, 16 m	56.14 (11.5)	ACT	CBT	Breast, Blood, Gastrointestinal, Lung, HN, Prostate or Testicular, Other	HADS-A: marginally more improvement in intervention group than control, CARS: significant better improvement in intervention group, IES: same	RCT	NCT02550925	US
2021	Kelleher et al. [53]	BPI, Self-Efficacy for Pain Management subscale of the Chronic Pain Self-Efficacy Scale, FACT-G, BSI-18	12 f, 19 m	59.5 (10.5)	Telephone-based coping skills training (CST) intervention for pain management	CBT	Colorectal	Self-efficacy for pain management and health-related QoL improved for all participants.	RCT (Pilot)	Not reported	US
2021	Hulbert-Williams et al. [91]	PHQ-9, GAD-7, FACT-G, SCNS SF-34, IES-R, SEMCD	32 f	IG: 44.50 (13.07), CG: 68.81 (9.52)	Low-intensity CBT intervention group delivered - 7 week (ACT)	CBT	Gynaecological	HADS: no changes related to CBT, improvement over time in both groups	RCT	NCT03553784	UK
2022	Henneghan et al. [54]	FACT-Cog; PROMIS-SF; Perceived Stress Scale; PROMIS Pain interference SF	31 f	52.22 (10.18)	Remote Mantra Meditation OR Music listening for 12 minutes a day	CBT (mind-body)	Breast	Short-term improvements in cognitive functioning and QoL	RCT	NCT03696056	US
2024	Wang et al. [108]	BPI, HADS, QOLCSV-C, FACT-B	68 f	Not reported	Breathing Exercise (BE) for chronic pain	CBT (mind-body)	Breast	BE: improved anxiety, depression, QoL all measures. 3 mild discomfort: dizziness, tiredness, yawning	RCT (Phase 2)	Not reported	Australia
2024	Hall et al. [55]	FCRI, CES, feasibility/acceptability scales	53 f, 11 h	52.4	IN FOCUS (8 sessions, mind-body resiliency) vs usual care	CBT (mind body)	Breast 45.3%, Haematological 17.2%, Genitourinary 12.5%, Gynaecological 7.8%, Sarcoma, Head/neck, Melanoma	Significant reduction in fear of recurrence (FCRI) at 2 months vs usual care (d=0.66), effect attenuated at 5 months; resilience (CES) increased at 5 months	RCT (Pilot)	NCT04876599	US
2025	Tran et al. [109]	FCRI-SF, FCR1r	41 f, 12 m	57.74 (15.03)	Stepped Care: Conquer Fear versus purpose-developed clinician-guided self-management intervention (FearLess)	CBT	Breast, Head and Neck, Gynaecological	FearLess: clinically significant improvement in FCR	Non-randomized pilot/feasibility	ACTRN12622000818730	Australia

2020	Kim et al. [103]	GPAQ, R-PFS-K, FACIT-B, HADS, PSQI, PSCS-8	48 f	IG: 49.91 (7.62), CG: 48.48 (6.75)	Better Life After Cancer: Energy, Strength, and Support (BLESS) exercise adherence program. Capital-based exercise adherence intervention (supervised and home-based exercise)	PA	Breast	BLESS: behavioural/severity domain significantly decreased, PA significantly increased. Both groups showed improvements in sleep quality, depression, anxiety, and QOL.	RCT	Not reported	South Korea
2021	Rutherford et al. [104]	EQ-5D, General Self-Efficacy Scale, FACIT-F, SPAQ, Qualitative data	121 f, 48 m	Not reported	CARE (Cancer and Rehabilitation Exercise)	PA	Prostate, Breast, Colorectal, Other	Improved physical function, independence, psychological well-being	Cohort Study (single-arm, pre versus post-interven-tion)	Not reported	Australia
2022	Macdonald et al. [105]	FACT-B, DASH, IPAQ	31 f	53.3 (10.8)	16-week classical ballet intervention	PA	Breast	Improved physical, psychological, social, cognitive wellbeing.	Cohort Study (single-arm, pre versus post-interven-tion)	Not reported	Australia
2023	Solk et al. [56]	PROMIS, ActiGraph GT3X-BT accelerometer (ActiGraph, Pensacola, Florida)	269 f	52.5 (9.9)	12 weeks, Fitbit + Fit2Thrive app and/or (i) support calls, (ii) deluxe app, (iii) text messages, (iv) online gym, and (v) buddy	PA	Breast	Improved Anxiety, Depression, Fatigue, Physical function, Sleep impairment, no change: sleep disturbance	RCT	NCT03131440	US
2023	Bantum et al. [57]	EORTC QLQ-C30, BFI, CES-D, Social Constraints Scale, FACT-cog, POMS	42 f	65	Traditional Hawaiian dance HULA	PA	Breast, Gynaecological	IG: slight improvement at T3 in depression, fatigue, health-related quality of life, mood, or social	RCT	NCT02351479	US
2023	Humphreys et al. [92]	SPAQ, FACIT, VAS, Self-efficacy	159 f, 93 m	57 (15.12)	City-wide physical activity pathway: the Active Everyday service	PA	Breast, Prostate, Colorectal, Head and Neck, NHL, Other	FACIT: decreased perceived fatigue. VAS: Improved PA. Self-efficacy: improved.	Cohort Study (single-arm, pre versus post-interven-tion)	Not reported	UK
2023	Beebe-Dimmer et al. [58]	FACT-G, FKH	25 f, 12 m	58.5 (10.5)	Cross-training & PA (CAPABLE), 12 weeks	PA	Breast, Prostate, Endometrial, Hodgkin's lymphoma, Kidney, Kidney & bladder	Improved QoL (all domains), improved emotional response to exercise	Non-randomised pilot/feasibility	NCT03750981	US
2023	Golsteijn et al. [87]	CIS, EORTC QLQ-C30, Wearable ActiGraph GT3X-BT, HADS	62 f , 416 m	66.5 (7.7)	Web based and Print based OncoActive versus control	PA	Prostate, Colorectal	OncoActive: lower fatigue, better physical functioning, no change in depression. Control: increased depression	RCT	Not reported	Netherlands
2024	Lee et al. [59]	IPAQ , GLTEQ, 30-STS, PROMIS-GH	585 f	59.3 (10.6)	ALAC: 12-session community-based program	PA	Breast (metastatic and early stage survivors)	IG: increased PA, improved physical and mental health	Cohort Study Pre-post, single-arm (with a within-cohort subgroup comparison)	Not reported	US
2024	Angelillo et al. [113]	PROMIS–29 Profile v2.1 , FCV-19S	10 f, 7 m	31.5 (7.3)	12-week personalised exercise prescription	PA	Lymphoma	Ability to participate in social roles/activities, sleep disturbance improved, decreased FCR (still clinically significant)	Non-randomised pilot/feasibility (Single-Arm Proof of concept)	Not reported	Canada
2024	Krell et al. [83]	EORTC QLQ-C30, FACT-F, ICD-10 criteria for cancer-related fatigue, IPAQ-L	37 f, 55 m	52.3 (16.5)	Telemedicine-based exercise intervention 6 months	PA	Prostate, Colorectal, Breast, Lymphoma, Leukaemia, Testicular, Bladder, Thyroid, Other	IG: improved QoL, fatigue, physical, social, cognitive functioning. Emotional functioning decreased.	non-RCT (pre versus post-intervention)	Not reported	Germany
2024	Takemura et al. [98]	PSQI, HADS, EORTC QLQ-C30, EORTC QLQ-LC13, BFI, Sleep time, disturbance, 6MWT	122 f, 104 m	61.41 (8.73)	Tai Chi versus Aerobic Exercise versus Control (16 weeks)	PA	Lung (Advanced included)	Aerobic Exercise (AE) and Tai Chi (TC) groups: improved sleep quality, psychological distress, physical function, step count, circadian rhythm. TC : greater improvement in sleep than AE.	RCT	NCT04119778	China
2025	Parker et al. [60]	GLTEQ, PASSS, PACES-8, BREQ-3, PSS-10, SF-36, BMI	17 f, 1 m	61.5	YMCA Pickle ball	PA	Breast, Haematological, Colorectal, Appendiceal, Lung, Melanoma, Pancreatic, Sarcoma, Thyroid	Significant improvement in physical activity, aspects of fitness, physical functioning, social support	Non-randomised pilot/feasibility	Not reported	US

2025	Millet et al. [93]	EORTC QLQ-30, HADS, Fitbit Wearable	30 f	40 (7.95)	PPA intervention (ACCEPTANCE)	PA	Gynaecological (Cervical)	Improved anxiety, depression, fatigue	Non-randomised pilot/feasibility	ISRCTN16349793	UK
2025	Bertoli et al. [116]	EORTC QLQ-C30, EORTC QLQ-BR23, SF-36	43 f	55.2 (7.6)	Mat Pilates	PA	Breast	IG: SF-36: improved physical limitations, mental health, emotional functioning, social functioning, general health.	RCT	Not reported	Brazil
2020	Cheon et al. [101]	Knowledge about survivorship and relevant lifestyle issues, HADS, FACT-B	97 f	52.7 (9.3)	Patient education session with trained nurse	PE	Breast	FACT-B: improved physical well-being and breast cancer sub-scale, HADS: improved anxiety and depression	Single Arm Trial	Not reported	South Korea
2020	Im et al. [61]	MSAS-SF, QASPB, Cancer Behaviour Inventory, PRQ-2000	115 f	51.11 (11.41)	Technology-Based Information and Coaching/Support Program	PE	Breast	Significant decreases in outcome variables, including pain and symptoms in both groups. More decreases in physical symptom distress scores in intervention group	Randomised Pre/Post-test Design	Not reported	US
2020	Manne et al. [62]	EORTC QLQ-HN35, 10-item scale on fostering preparedness, for the study created questionnaires	27 f, 39 m	63.2 (9.5)	Empowered Survivor: e-Health self-management intervention	PE (eHealth)	Oral, Oropharyngeal	Reduced information and support needs; improved HrQoL (lower scores over time), with significant improvements in dry mouth, mouth opening ability, social contact, sensory problems, and speech difficulties..	Single-arm pilot study	Not reported	US
2020	Ashing et al. [63]	FACT-G	40 f	Not reported	Telephone PE	PE	Breast	Higher emotional wellbeing in the intervention group	Randomised Comparison (secondary analysis)	Not reported	US
2021	Im et al. [64]	CBI, SCNS-SF34, PIS, PRQ-2000	67 f	52.50 (12.00)	Technology-based program (social media, educational modules, online resources)	PE	Breast	Increases self-efficacy in CG and IG. Changes lasted over time only in intervention group.	RCT	Not reported	US
2022	Syrjala et al. [65]	CTXD, PHQ-8, FSI, BPI, ISQ, Confidence in survivorship information	242 f, 102 m	35.40 (5.70)	Survivorship program (PE and feedback on psychosocial evaluation)	PE	Breast, Leukaemia, Lymphoma, Other solid tumours not specified	IG: decreased distress	RCT	NCT02192333	US
2022	Singleton et al. [106]	EORTC QLQ-C30, EORTC QLQ-BR23, SEMCD, GPAQ, ActiGraphTM GT3X+ (ActiGraph, Pensacola, FL)	156 f	54.8 (10.9)	EMPOWER-SMS: text messages on various topics	PE	Breast	Six-month follow-up: no significant differences between IG and CG on QoL	RCT	Not reported	Australia
2023	Sohl et al. [66]	Acceptability measures (diverse), SEMCD, PROMIS Profile 29, Nutrition, mindfulness meditation practice,	29 f, 11 m	57.4	Shared Healthcare Actions & Reflections Electronic systems in Survivorship (SHARE-S)	PE (Coaching)	Breast, Gynaecologic, Prostate, Other not specified	Improvements in mindful attention, lifestyle behaviour, depressive symptoms, ability to participate in social roles, cancer-specific QoL, positive feelings	Non-randomised pilot/feasibility	NCT04337203	US
2023	Wang et al. [67]	EORTC QLQ-C30, EORTC QLQ-CR29	23 f, 17 m	55	12-week web-based dietary intervention with text messages	PE	Colorectal	IG: decreased fatigue, no other QoL changes	RCT	NCT02965521	US
2024	Gutnik et al. [68]	CLSS, Breast cancer knowledge assessment	30 f	55 (16.224)	Survivor-led Stigma reduction intervention (SRI) (Education Flip Chart, Testimony, MI)	PE	Breast	Decreased Stigma post intervention. Survivors felt "empowered" and "valued"	Cohort Study (single-arm, pre ver-sus post-interven-tion)	Not reported	US/Tanzania
2024	Heneka et al. [107]	eHEALS, EPIC, DT and Problem List validated for prostate cancer, ISI, BFI, GSLTPAQ, EQ5D-5L	17 m	64.0 (5.0)	12-week virtual care program delivered via teleconference by a specialist nurse	PE	Prostate	DT: decreased distress (less problems urinary function, pain, work, uncertainty regarding future). EPIC: decreased symptom burden, insomnia.	Non-randomised pilot/feasibility	Not reported	Australia
2024	Berzins et al. [69]	IPAQ, REAP-S, PSQI, PSS, HADS, FCAT-G	37 f, 11 m	Not reported	Community-Based Wellness Coaching	PE	Breast, NHL, Ovarian, Lung, Thyroid, Melanoma, Endometrial, Multiple Myeloma, Prostate, Leukaemia, Hodgins Lymphoma, Appendix,	PSS: reduced perceived stress, PQSI: improved sleep, HADS: decreased anxiety and depression, FACT-G: improved well-being	Cohort Study (single-arm, pre versus post-interven-tion)	Not reported	US

							Colorectal, Sinus paraforma, Throat, GIST, Testicular, Kidney, Kidney				
2020	Ji et al. [100]	FACT-B	118 f	48.0 (8.8)	Expressive Writing (SR Self-regulation group, EMO emotional disclosure group, CTL cancer-facts writing group, and CON control group)	Psychosocial & Self-directed support (Art therapy)	Breast	CTL, EMO, SR groups higher levels of overall QoL, higher physical wellbeing, emotional well-being, than the control group. CTL group higher physical well-being and EMO group higher social well-being than the SR group.	Randomised Trial	ChiCTR1900022045	China
2021	Thompson et al. [70]	RAND 36-Item Health Survey, CES-D, CARS, FACT-B	228 f	IG: 55.8 (9.7), CG: 56.0 (10.3)	Breast Cancer Survivor Stories Intervention: tablet-computer with survivor stories three times in 12 months	Psychosocial & self-directed support (Peer Support)	Breast	IG: no improvements. Longer use: negative impact FCR, emotional wellbeing, energy/ fatigue, role functioning	RCT	NCT00929084	US
2023	Boland et al. [84]	FAI, HADS, FACIT-F, COPM, SES, EQ-5D-3L QoL health states index score	73 f, 7 m	51.68 (11.73)	OptiMal : group based	Psychosocial & self-directed support (Self-Management)	Breast, Other not specified	HADS: decreased anxiety in IG, COPM: improved performance IG.	RCT	Not reported	Ireland
2023	Tauber et al. [82]	FCRI, DASS–21, EORTC QLQ-C30, SUNS, DERS, FFMQ, EQ, MCQ-30, PSWQ, RRS–B, NEQ, WAI-SR, TFI-CS	85 f	Not reported	ConquerFear: online-delivered group-based psychological intervention, versus relaxation training	Psychosocial & self-directed support (Psychological, mind-body)	Breast	IG reduced FCR, FCR severity. Improved mindfulness, decentring, emotion regulation, worry, rumination, compared to CG.	RCT	NCT04137575	Denmark
2023	Zhao et al. [96]	FCRI, EORTC QLQ-C30, DASS-21	26 f, 55 m	CALM 62.05+/-8.68 years; UC 64.29+/-9.33 years	CALM	Psychosocial & self-directed support (emotion focused)	Lung	FCR significantly reduced in the CALM group vs UC (F=315.20, p<0.001); general distress (DASS-21) significantly reduced (F=290.10, p<0.001); QOL (EORTC QLQ-C30) significantly improved (F=220.30, p<0.001) at T1/T2/T3; negative correlations between NLR-QOL, FCR-QOL and distress-QOL	RCT	Not registered	China
2024	Merckaert et al. [81]	HADS, FCRI-SF, PSWQ, WBSI	120 f	53.8 years	Group intervention on emotion regulation and self-regulation, eight 2.5-hour sessions, ecologically boosted with daily app-based prompts (LifeData) vs delayed arm (intervention 5 months later)	Psychosocial & self-directed support (Hypnosis, Emotion regulation)	Breast	Significant reduction in worry and intrusive thoughts; improved ability to down-regulate negative emotions; daily negative emotions less frequent/intense, daily positive emotions more frequent; benefits maintained at 5 months except for daily positive emotions.	RCT	NCT03336827	Belgium
2024	Du et al. [97]	FCRI, STAI,KPS, Cortisol, Heart Rate	15 f, 47 m	57	Music Therapy (without) and (with therapist)	Psychosocial & self-directed support (Art therapy)	NSCLC	Reduced anxiety in Intervention Group (with therapies)	RCT	ISRCTN23276302	China
2024	Le et al. [99]	DASS-21, SF-12, CBI-B, MOS-SSS	186 f	49.26 (11.92)	Stronger Together Peer Mentoring Model	Psychosocial & self-directed support (Peer Support)	Breast, Gynaecological	IG: improved depression, anxiety, stress, mental health, depression, social support, QOL (improvements later and less in CG).	Non-randomised pilot/feasibility	Not reported	Vietnam
2024	Vagnini et al. [85]	BIS, Brief-Cope, RSES, GSE	40 f	IG: 56.55 (11.48), CG: 52.40 (9.93)	Photography session & narrative session psychotherapist versus waitlist	Psychosocial & self-directed support (Art therapy)	Breast	IG: Improved body image, problem focused coping, emotion-focused coping, self-efficacy	Non-randomised pilot/feasibility	Not reported	Italy
2025	Kim et al. [102]	CS-SES-K, K-Mini-MAC, WHOQOL-BREF, PHQ-9, GAD-7), MESQ, for the study created 17-item satisfaction questionnaire	72 f	IG: 55.2 (7.8), CG: 52.3 (8.8)	CAMA: Mobile App for Breast Cancer Self-Management on Self-Efficacy 12 weeks (versus treatment as usual)	Psychosocial & self-directed support	Breast	IG: improved help-seeking and support, positive attitude, psychological well-being. Reduced anxiety.	non-RCT, Intervention vs. non-intervention (treatment-as-usual control, non-randomized)	KCT0007917	South Korea

2021	Walker et al. [112]	SDS, VSQ, Vaginal Health Promotion Strategies Questionnaire, IIEF, RDAS, PHADS	59 f, 18 m	51.00 (11.5)	Oncosexual Health Clinic: multidisciplinary team of psychologists, advanced practice nurses, and radiation and gynaecological oncologists	Functional & multidisciplinary care (Integrated)	Hematologic, Breast, Gynaecologic, Gastrointestinal, Genitourinary, Sarcoma, Other (lung)	Significant decrease in sexual distress	Non-randomised pilot/feasibility	Not reported	Canada
2022	Polo et al. [71]	COPM, PROMIS, PSQI, MAF	26 f, 9 m	65,1	OT-HAWP (Occupational Therapy Health and Wellness Program) 4-week community program, six 90-min weekly sessions	Functional & multidisciplinary care (Occupational therapy, PE)	Gastrointestinal, Gynaecologic, Leukaemia/Lymphoma, Breast, Skin, Other Not reported	Significant improvement on all outcomes, COPM-Performance, Satisfaction (p=.001, d=1.28), PROMIS Physical Health , PROMIS Mental Health, PSQI sleep quality, MAF fatigue	Non-randomised pilot/feasibility	NCT03888547	US
2024	Areklett et al. [89]	HADS, EORTC QLQ-C30, Objective cognitive functioning CANTAB®, BRIEF-A	35 f	49.7 (7.2)	eHealth training intervention	Functional & multidisciplinary care (Cognitive training)	Cervical	IG: less fatigue than control group at T2 + better social functioning. Anxiety decreased over time in both groups.	Randomised Comparison	Not reported	Norway
2021	Blair et al. [72]	Social Provisions Scale, PROMIS, Sedentary Behaviour Questionnaire, Device measures	21 f, 9 m	68	Harvest for Health : home-based vegetable gardening	Gardening	Breast, Prostate, Lung, Other	PROMIS: improvement in social functioning and well-being	Single Arm trial	Not reported	US
2020	Jammu et al. [111]	DT, Canadian Problem Checklist (CPC), ESAS	155 f, 21 m	Not reported	Survivorship Clinic	Mixed (PE, Peer support, …)	Breast, Other not specified	DT: Improved. Edmonton Symptom Assessment Scale: significant decline in reported pain, tiredness, nausea, depression, anxiety, drowsiness, shortness of breath scores	Retrospective Study (Data analysis) (pre versus post-interven-tion)	Not reported	Canada
2020	Ho et al. [94]	SF-12, FACT-C, HADS	82 f, 141 m	65.2 (10.1)	Dietary and PA interventions	Mixed (PA, PE)	Colorectal	SF-12 PCS: Improvement in QoL FACT-G: Improvement in QoL (total score). HADS: No changes in anxiety between groups. Significant improvement in depression in dietary intervention group.	RCT	NCT01708824	China
2020	Dhruva et al. [73]	EORTC QLQ-C30, EORTC QLQ-BR23, STAI-S, STAI-T, GSDS, LFS, CES-D, PSS, FACIT-SP	32 f	48.0 (10.0)	Whole-Systems Ayurvedic Medicine Nutrition and Lifestyle Intervention	Mixed (Counselling, PE, PA (Yoga))	Breast	Improved emotional, cognitive, social and global health functioning, fatigue, insomnia, and sexual enjoyment (EORTC QLQ-C30); improved sleep quality, reduced sleep disturbances and daytime sleepiness (GSDS); reduced fatigue (LSF), depressive symptoms (CES-D), anxiety (STAI-S), and perceived stress (PSS); improved spiritual well-being (GSDS).	Single Arm Pilot Trial	Not reported	US
2020	Galantino et al. [74]	BPI, PNQ, FACT-GOG-Ntx, PTSQ, FACIT-Sp, Fear of falling (FES)	7 f, 1 m	65	Somatic Yoga, Meditation	Mixed (PA, mind-body)	Breast, Colon, Ovarian, Pancreatic	FACT-COQ-Ntx, PNQ, FES: improved symptoms; PSS: reduced stress; PSQI: improved sleep, FACT-SP: improved spirituality	Pilot Study	Not reported	US
2021	Tack et al. [80]	CFQ, DT and 38-item problem list (PL), BDI-II, FACIT-F, EORTC QLQ-C30, EuroQol EQ-5D-3L	113 f, 8 m	52.2 (8.0)	Emotional Freedom Techniques (Acupressure, CBT, Neurolinguistic reprogramming)	Mixed (CBT, CAM)	Breast, NHL, Gynaecological	IG: CFQ reduced, distress, depressive symptoms, fatigue and improved QoL compared to CG.	RCT	NCT02771028	Belgium
2022	Langlais et al. [86]	EORTC QLQ-C30, PROMIS Fatigue 8a – Adult v1.0, PSQI	202 m	70	Remotely Delivered Web-Based Behavioural Intervention	Mixed (PE, Coaching, CBT)	Prostate	No significant impact	RCT (Pilot)	NCT03406013	Netherlands
2022	Tami-Maury et al. [75]	PROMIS, 6MWT, International Physical Activity Questionnaire (SF)	187 f	59.6 (10.7)	12 weeks: PA, psychoeducation and CBT on various topics	Mixed (PA, PE, CBT)	Breast	Significant improvement in physical and mental health domains	Cohort Study	Not reported	US

2022	Medina et al. [90]	HADS, PCL-5, PTGI, EQ-5D-3L, MOS-SSS	189 f	52.3	ICOnnecta't stepped eHealth ecosystem (screening/monitoring, PE campus, peer-support community, group psychotherapy)	Mixed (eHealth / Stepped psychosocial care)	Breast	Distress (HADS) within expected/moderate range; QoL (EQ-5D-3L) below population cut-off at baseline, no significant change over time; strong social support associated with better psychosocial course	Non-randomised pilot/feasibility	Institutional registry PR343/18	Spain
2023	Sun et al. [95]	SAS, SDS, FCRI, EORTC QLQ-C30, Gut microbiom analysis 16Sr RNA gene sequence	14 f, 24 m	58.00 (13.00)	TCM + online group therapy intervention 6-week	Mixed (Psychotherapy, CAM)	Colorectal	Decreased FCR, Depression. QLQ-C: improvement in symptom burden of insomnia	Single-arm Trial (Phase 1)	Not reported	China
2023	Zimmerman et al. [76]	FACIT-F, PSQI, FSI, MAIA, DERS, MSPSS, PSS, Unmitigated Communion (UC), BRS, PHQ, SF-36	24 f	57.30 (9.00)	Qigong and Exercise/ Nutrition Training	Mixed (PA, PE)	Breast, Gynaecological, Kidney, Colorectal, Thyroid, Multiple myeloma, Melanoma	Both groups improved fatigue, without significant difference. QIGONG: improved mood, emotion regulation, and stress	RCT (Pilot)	Not reported	US
2024	Allison et al. [77]	PHQ-9, City of Hope Breast Cancer QoL Scale , IPAQ	21 f	60.7 (11.6)	Group behavioural Weight loss	Mixed (Peer Support, CBT)	Breast	PHQ-9 : Mood improved, depressive symptoms reduced, QOL-BC Scale: physical well-being improved. No other changes.	Non-randomised pilot/feasibility	Not reported	US
2024	Crespi et al. [78]	Work Productivity and Activity Impairment scale	246 f	45.4 (6.4)	Pathways to Wellness	Mixed (PE, CBT (mind-body))	Breast	Reduced stress, fatigue, work productivity loss and depression	RCT	NCT03025139	US
2024	Ilie et al. [114]	K10	104 f	With 60, Without: 57	Empowerment Program (with and without HeartMath biofeedback device) Home based	Mixed (PA, PE)	Bladder, Blood, Breast, Gynaecological, Colorectal, Kidney, Lung, Pancreatic, Skin	Significant reduction of distress in both groups. No significant impact of HRV biofeedback on distress.	RCT	NCT05508412	Canada
2024	Tyrus et al. [79]	CES-D, GAD-7, PHQ-8,	247 f	45	Survivorship Education (SE, 6 weekly 2h group sessions) vs Mindful Awareness Practices (MAPs, 6 weekly 2h group sessions) vs waitlist control (WLC)	Mixed (PE OR CBT (mind-body))	Breast	Baseline depression and anxiety moderated SE effects: participants with higher symptoms and lower survivorship preparedness showed greater depressive symptom reductions after SE. MAPs benefits were independent of baseline characteristics; neither intervention preference nor time since diagnosis moderated effects.	RCT	NCT03025139	US
2025	Carlson et al. [115]	PHQ-9, GAD-7, FACT-G, SCNS SF-34), IES-R, 11-, EQ-5D VAS, qualitative interviews - plus the Profile of Mood States (POMS)	440 f, 146 m, 1 Not reported	60.7 years	Mindfulness-Based Cancer Recovery (MBCR) vs Tai Chi/Qigong (TCQ), preference-based design + 2:1 random assignment (immediate intervention vs waitlist)	Mixed (mind-body) OR PA	Mixed types (12 types) - breast 40.7%, prostate 11.2%, gastrointestinal 9.7%, other	Both MBCR and TCQ improved mood vs waitlist regardless of allocation; TCQ reduced depression/anger, MBCR reduced tension-anxiety, both increased vigour, with greater benefits in women (MBCR) and younger patients (TCQ).	RCT	NCT02801123	Canada

[1] Reports main findings regarding the different studies. Entries are regrouped by type of intervention and in chronological order. A colour code has been attributed to the variable “intervention type” which is green for physical activity (PA), blue for cognitive behavioural therapy (CBT), orange for Psychoeducation (PE), pink for other types of psychosocial or self-directed support, yellow for interventions consisting in various components and therefore having not been attributed to one of the other categories, lavender for functional and multidisciplinary interventions, and white for a gardening intervention which did not fit any of the other categories.

Table S2. Summary of results extracted from systematic literature reviews and meta-analysis.

Year	Author	Measures*	Participants	Intervention	Intervention Type	Cancer	Psychosocial Outcomes	Study Type	Prospero/review registration	Studie (N)	Type of studies	Data bases	Search Period
2020	Hwang et al. [37]	ACT, BFI, BL, BMI, BPI, BSI-GSI, CES-D, CI, CIS, CSD, CTXD, DBAS-16, DQI, EMA, EORTC QLQ-C30, EPIC, FACIT-F, FACIT-Sp-Ex, FACT-B, FACT-Cog, FACT-G, FSI, F&V, HADS, IG, IPAQ, ISI, IT, LD, MRS, NA, NPS, PA, PEPPI, PSQI, QOL, RDA, RPE, R-PFS, RR, SCL-90-R, SE, SF-36, SEPA, SOL, DT, TIB, TMT, TST, WASO, WEL, WL, 6MWT	2688	Technology-Based Telehealth Approach for Occupational Therapy Interventions	eHealth, CBT, PE	Breast (75%), Gynaecological, Prostate, Hematological	Symptom self-management reduced symptom burden. Psychosocial interventions (e.g., CBT, problem-solving, CBT-I, mind–body training) reduced sleep disturbance and improved PA. Lifestyle interventions improved PA and diet quality while reducing pain, depression, fatigue, and distress, and improving quality of life.	SR	Not reported	15	RCT, quasi-experimental Study	Medline Complete, PubMed, CINAHL, PsycINFO, Web of Science	inception - 2020
2021	Mathew et al. [38]	WAI-SR, MCQ30, AQOL-8D, FCRI, PROMIS, Cancer AAQ, GAD, PHQ-8, PHQ-4, PSS, PSQI, SPS, FACT-O, BADS, HADS, MCQ30, DASS21, IES, IBQ, RAND SF-36, STAI, CES-D, BDI-II, BAI	537 (gender not specified)	ACT (Acceptance and Commitment Therapy)	Psychotherapy, CBT	Breast, Colorectal, Melanoma, Other not specified	ACT significantly reduced anxiety, depression, and FCR and improved psychological flexibility and QoL.	SR	Not reported	13	experimental (including pilot and single-arm studies)	CINAHL, MEDLINE via Ovid, Web of Science, PsycInfo, Scopus, Embase, Google Scholar, and Cochrane databases	2015-2019
2021	Ratcliff et al. [39]	Coping, POMS, MOS, PCL-S, MAC, COPE, Side-effect diary, STAI, EORTC QLQ-BC23, SF-36, FACT-B, HADS, Health behaviours, CES-D	female (number not reported)	Psychosocial Support, Weight management, Psycho-education	CBT	Breast	Six out of eight intervention conditions reported favourable outcomes compared to control conditions, suggesting promise for efficacy.	SR	Not reported	19	RCT, quasi-experimental Study	MEDLINE, Ovid Embase, PubMed and PsycInfo	2000-2020
2022	Margalit et al. [40]	MBOE, DAS-59, SWAL-QOL, SHI, SDQ, PAM, EORTC QLQ-C30, EORTC QLQ-H&N35, FACT-An, NDII, SWSF, XI, OHIP-14, CPPS, GRBAS, XeQoLS	2061 (gender not specified)	Resistance training, chewing gum, Self-care education, Voice rehabilitation, Stepped care program for psychosocial distress, Medical interventions	PE, Rehabilitation, PA	Head and Neck	Psychosocial outcomes were mixed: most interventions showed no significant effects, while some reported improvements in self-efficacy, depression, unmet needs, and survivorship knowledge. Evidence remains limited and heterogeneous.	SR	CRD42021269566	28	RCT, non-RCT	MEDLINE, Embase, Emcare, and PsycINFO	2000-2021
2024	Giridharan et al. [41]	PSQI, Actigraphy, MFSI-SF, Clinical symptom inventory, FACIT-F, MDASI, General linear modelling	Not reported	Yoga for Cancer Survivors (YOCAS)	PA	Breast 65-100% (other not specified)	YOCAS program effectively reduced cancer-related fatigue, improved sleep quality, and addressed the cognitive and musculoskeletal symptoms	SR	Not reported	6	RCT	Google Scholar, Scopus, Cochrane Library, PubMed	2000-2024
2020	Armer et Lutgendorf [42]	Not reported	1828 (gender not specified)	Yoga	PA	Breast Cancer (72%), Colorectal, Prostate	Physical Yoga: decreased fatigue, Yoga: decreased depression, No changes in QoL	MA	Not reported	55	RCT	PubMed, PsycINFO	2007-2018
2022	Addison et al. [43]	CIS-Fatigue, Sickness Impact Profile, PSOM, ABS, MOCS, POMS-F/I, STAI, CES-D, FACT-B, ISI, Geriatric Depression Scale-Short Form, PSQI, Epworth Sleepiness Scale, HADS, FSI, FACT, MASQ, PCI, SIP-8, SCL-90, SF-36, EORTC QLQ-C30, HAM-A, CGI, MADRS, PSS, IES,	5199 (3629 f, 1282 h, 57 not specified)	CBT and Healthy lifestyle intervention	CBT	Breast, Head and Neck, Colorectal, Gynaecological, Testicular, Other solid tutors, Sarcoma, Melanoma, Thyroid, Urogenital, Lymphoma, Pancreatic, Lung	Most studies (31 of 36) reported a reduction in adverse treatment and/or cancer-related effects.	MA	Not reported	36	RCT	PubMed, PsychINFO, CINAHL, Embase	1990-2019

		LOT, RAND-36, Revised Piper Fatigue Scale, WHQ, EPIC, SES											
2023	Lui et al. [44]	Not reported	120 (not specified)	Various	PE, Psychotherapy, Support Group, Self-regulation, Mind-body	Breast, Colorectal, Prostate, Gynaecological	The interventions under review had small but beneficial effects on psychosocial outcomes for immigrant and LEP cancer survivors.	MA	CRD42020213669	45	RCT, non-RCT, single-arm trial	PubMed, Embase, Cochrane Central Register of Clinical Trials (Cochrane/Wiley), PsycINFO, CINAHL	inception-2022
2024	Abdul Razak et al. [45]	FACT-F, FACT-C, PSQI, Centre for Epidemiological Studies Depression scale, Patient Health Questionnaire, HADS, STAI	f 234, m 195	PA, PE (nutrition)	PA, PE	Colorectal	No significant effect of exercise intervention on HrQoL in the intervention group compared to control group	MA	CRD42022332393	7	RCT	EBSCOhost, Web of Science (WOS), Scopus, Science Direct, PubMed	2003-2020
2024	Dils et al. [46]	Multiple	13.226 (female (n = 10,303)		CBT	Multiple	CBT moderately improves MH and QoL in cancer survivors, age and delivery format can influence the efficacy of CBT in this patient population.	MA	CRD42020200987	132	Clinical Trial	11 databases, and 4 websites (not specified)	1986-2023
2024	Gray et al. [47]	FACIT-F	96 males, 229 females	Resistance Training	PA	Breast, Gynaecological, Prostate	Main findings indicate that RT ameliorates CRF, especially in BCS	MA	Not reported	8	RCT	MEDLINE, CINAHL via Ebsco, EMBASE, Cochrane Library, PubMed, Scholar	inception-2022
2024	Telles et al. [48]	PTGI, PTGI-SF, PROMIS-SRS, PROMIS-ES, PROMIS-PAW, SCS/SCS-SK, FACT-SWB, FACT-EWB, WHOQOL-BREF, WHO-5, LOT-R, LiSat-9, PERMA-Profiler, Satisfaction with Life Scale, PANAS, FACIT-M/P, FACIT-Sp, PSOM, PCQ, MHC-SF, MHS	2651 (2200f, 451 h)	ACT, MBCT, MBSR, Meditation	Mindfulness, CBT, Psychotherapy	67.7% of studies (n = 21) focused on only one diagnosis (17 breast, 1 prostate, 1 brain, 1 melanoma, 1 cervical); 32.3% (n = 10) included multiple or mixed diagnoses.	Mindfulness-based interventions improved eudemonic, hedonic, and social well-being vs controls (SMD = 0.599), regardless of intervention characteristics, with a trend toward greater effects in diagnostically homogeneous than heterogeneous groups (SMD = 0.66).	MA	Not reported	31	RCT	PubMed, PsycInfo, Web of Science, ProQuest Dissertations & Theses Global, CINAHL, Scopus	inception-2022
2024	Coyne et al. [49]	HADS, SF-12, PSS, heiQ v3.0, HLQ, IPAQ-SF, DCI, RDAS, System Usability Scale, EQ-5D-5L, FACT, Distress Thermometer, Charlson Comorbidity Index, CHAMPS questionnaire, SF-36, body composition, PPB	704 survivors, 411 other participant groups (gender not specified)	TEMPO (web-based dyadic psychosocial/PA intervention), Exercising Together (couple-based resistance training), LifeChamps (co-designed digital platform), and descriptive/qualitative studies on supportive care needs, sexuality, and cognition.	PE, PA, eHealth	Prostate, Brain, Lymphoma, Other (non specified)	Clinically significant improvement in anxiety and quality of life (QoL) (TEMPO); physical and psychological benefits of couple-based strength training; unmet supportive care needs identified in communication, emotional support, sexuality/intimacy, and cognitive functioning.	Integrative Review	CRD42021229080	7	2 RCT, 3 observational studies, 2 qualitative studies	Medline (PubMed), CINAHL, Cochrane Library, LILACS	2012-2024

[2] MA are highlighted in green, SA in blue, and an additional type of review in grey.

The following abbreviations (mainly of measures and instruments) are used in the tables:
6MWT, 6-Minute Walk Test; 30-STS, 30-Second Sit-To-Stand Test; ABS, Affect Balance Scale; ACT, Auditory Consonant Trigrams; AQOL-8D, Assessment of Quality of Life-8D; BADS, Behavioral Activation for Depression Scale; BAI, Beck Anxiety Inventory; BDI-II, Beck Depression Inventory-II; BFI, Brief Fatigue Inventory; BIS, Body Image Scale; BPI, Brief Pain Inventory; BREQ-3, Behavioral Regulation in Exercise Questionnaire-3; BRIEF-A, Behavior Rating Inventory of Executive Function - Adult version; Brief-COPE, Brief COPE Inventory; BRS, Brief Resilience Scale; BSI, Brief Symptom Inventory (BSI-18, 18-item version; BSI-GSI, Global Severity Index); Cancer AAQ, Cancer-specific Acceptance and Action Questionnaire; CANTAB, Cambridge Neuropsychological Test Automated Battery; CARS, Concerns About Recurrence Scale; CBI, Cancer Behavior Inventory (CBI-B, Brief version); CES-D, Center for Epidemiologic Studies Depression Scale; CFQ, Cognitive Failures Questionnaire; CGI, Clinical Global Impression scale; CIS, Checklist Individual Strength (CIS-Fatigue, Fatigue Severity subscale); City of Hope Breast Cancer QOL Scale, City of Hope Quality of Life Scale - Breast Cancer version; COPE, COPE Inventory; COPM, Canadian Occupational Performance Measure; CPC, Canadian Problem Checklist; CS-SES-K, Cancer Survivorship Self-Efficacy Scale - Korean version; CSD, Consensus Sleep Diary; CTXD, Cancer and Treatment Distress scale; DAS-59, Derriford Appearance Scale-59; DASH, Disabilities of the Arm, Shoulder and Hand questionnaire; DASS-21, Depression Anxiety Stress Scales-21; DBAS-16, Dysfunctional Beliefs and Attitudes about Sleep-16; DCI, Dyadic Coping Inventory; DERS, Difficulties in Emotion Regulation Scale; DQI, Diet Quality Index; DT, Distress Thermometer; eHEALS, eHealth Literacy Scale; EORTC QLQ, European Organisation for Research and Treatment of Cancer Quality of Life Questionnaire (-C30, Core 30; -BR23, Breast Cancer Module, 23 items; -CR29, Colorectal Cancer Module, 29 items; -H&N35, Head and Neck Cancer Module, 35 items; -LC13, Lung Cancer Module, 13 items); EPIC, Expanded Prostate Cancer Index Composite; EQ-5D, EuroQol 5 Dimensions (EQ-5D-3L, 3-level version; EQ-5D-5L, 5-level version); ESAS, Edmonton Symptom Assessment System; FACIT, Functional Assessment of Chronic Illness Therapy (FACIT-F, Fatigue; FACIT-Sp, Spiritual Well-Being; FACIT-Sp-Ex, Spiritual Well-Being Expanded; FACIT-M/P, Spiritual Well-Being Meaning/Peace subscale); FACT, Functional Assessment of Cancer Therapy (FACT-G, General; FACT-An, Anemia; FACT-B, Breast; FACT-C, Colorectal; FACT-O, Ovarian; FACT-Cog, Cognitive Function; FACT-EWB, Emotional Well-Being subscale; FACT-SWB, Spiritual Well-Being subscale; FACT-F, Fatigue subscale; FACT-GOG-Ntx, Gynecologic Oncology Group Neurotoxicity subscale); FAI, Fatigue Assessment Inventory; FCRI, Fear of Cancer Recurrence Inventory (FCRI-SF, Short Form; FCR1r, single-item revised version); FCV-19S, Fear of COVID-19 Scale; FES, Falls Efficacy Scale; FFMQ, Five Facet Mindfulness Questionnaire; FSI, Fatigue Symptom Inventory; GAD-7, Generalized Anxiety Disorder-7; GPAQ, Global Physical Activity Questionnaire; GRBAS, Grade, Roughness, Breathiness, Asthenia, Strain voice quality scale; GSE, General Self-Efficacy Scale; GSLTPAQ, Godin-Shephard Leisure-Time Physical Activity Questionnaire; HADS, Hospital Anxiety and Depression Scale (HADS-A, Anxiety subscale); HAM-A, Hamilton Anxiety Rating Scale; heiQ v3.0, Health Education Impact Questionnaire, version 3.0; HLQ, Health Literacy Questionnaire; IBQ, Illness Behaviour Questionnaire; IES, Impact of Event Scale (IES-R, Revised); IIEF, International Index of Erectile Function; IPAQ, International Physical Activity Questionnaire (IPAQ-L, Long form; IPAQ-SF, Short Form); ISI, Insomnia Severity Index; K10, Kessler Psychological Distress Scale (10 items); K-Mini-MAC, Mini-Mental Adjustment to Cancer scale - Korean version; KPS, Karnofsky Performance Status; LiSat-9, Life Satisfaction Questionnaire (9 domains); LOT, Life Orientation Test (LOT-R, Revised); MAC, Mental Adjustment to Cancer scale; MADRS, Montgomery-Asberg Depression Rating Scale; MAF, Multidimensional Assessment of Fatigue; MAIA, Multidimensional Assessment of Interoceptive Awareness; MASQ, Mood and Anxiety Symptom Questionnaire; MCQ-30, Metacognitions Questionnaire-30; MDASI, MD Anderson Symptom Inventory; MFSI-SF, Multidimensional Fatigue Symptom Inventory - Short Form; MHC-SF, Mental Health Continuum - Short Form; MOCS, Measure of Current Status; MOS, Medical Outcomes Study health survey (MOS-SSS, Social Support Survey); MRS, Menopause Rating Scale; MSAS-SF, Memorial Symptom Assessment Scale - Short Form; MSPSS, Multidimensional Scale of Perceived Social Support; NDII, Neck Dissection Impairment Index; NPS, Net Promoter Score; OHIP-14, Oral Health Impact Profile-14; PACES-8, Physical Activity Enjoyment Scale (8 items); PAM, Patient Activation Measure; PANAS, Positive and Negative Affect Schedule; PASSS, Physical Activity-Specific Social Support scale; PCI, Perceived Cognitive Impairments subscale (FACT-Cog); PCL-5, PTSD Checklist for DSM-5; PCL-S, Posttraumatic Stress Checklist - Stressor-Specific version; PCQ, Psychological Capital Questionnaire; PEPPI, Perceived Efficacy in Patient-Physician Interactions; PERMA-Profiler, PERMA-Profiler (Positive emotion, Engagement, Relationships, Meaning, Accomplishment); PHADS, Perceived Health and Daily Symptoms; PHQ, Patient Health Questionnaire (PHQ-4, PHQ-8 and PHQ-9, 4-, 8- and 9-item versions); PIS, Pain Impact Scale; POMS, Profile of Mood States (POMS-F/I, Fatigue/Inertia subscale); PPB, Physical Performance Battery; PROMIS, Patient-Reported Outcomes Measurement Information System (PROMIS-ES, Emotional Support; PROMIS-GH, Global Health; PROMIS-PAW,

Psychosocial Illness Impact - Positive; PROMIS-SRS, Social Roles / Social Isolation domain); PRQ-2000, Personal Resource Questionnaire-2000; PSOM, Positive States of Mind scale; PSQI, Pittsburgh Sleep Quality Index; PSS, Perceived Stress Scale (PSS-10 and PSS-14, 10- and 14-item versions); PSWQ, Penn State Worry Questionnaire; PTGI, Posttraumatic Growth Inventory (PTGI-SF, Short Form); RAND-36, RAND 36-Item Health Survey; RDAS, Revised Dyadic Adjustment Scale; REAP-S, Rapid Eating Assessment for Participants - Shortened version; R-PFS, Revised Piper Fatigue Scale (R-PFS-K, Korean version); RPE, Ratings of Perceived Exertion scale; RRS-B, Ruminative Responses Scale - Brief version; RSES, Rosenberg Self-Esteem Scale; SAS, Self-Rating Anxiety Scale; SBQ-R, Suicide Behaviors Questionnaire - Revised; SCL-90, Symptom Checklist-90 (SCL-90-R, Revised); SCNS-SF34, Supportive Care Needs Survey - Short Form 34; SCS, Self-Compassion Scale (SCS-SK, Korean version); SDQ, Shoulder Disability Questionnaire; SDS, Self-rating Depression Scale; SEMCD, Self-Efficacy for Managing Chronic Disease 6-Item Scale; SEPA, Self-Efficacy scale for Physical Activity; SF-12 and SF-36, 12- and 36-Item Short Form Health Survey; SHI, Speech Handicap Index; SIP-8, Sickness Impact Profile-8; SPAQ, Seasonal Pattern Assessment Questionnaire; SPS, Social Provisions Scale; SQUASH, Short Questionnaire to Assess Health-enhancing physical activity; STAI, State-Trait Anxiety Inventory (STAI-S and STAI-T, State and Trait subscales); SUNS, Survivor Unmet Needs Survey; SWAL-QOL, Swallowing Quality of Life questionnaire; TMT, Trail Making Test; VAS, Visual Analogue Scale; VSQ, Vaginal Symptoms Questionnaire; WAI-SR, Working Alliance Inventory - Short Revised; WBSI, White Bear Suppression Inventory; WEL, Weight Efficacy Lifestyle Questionnaire; WEMWBS, Warwick-Edinburgh Mental Well-being Scale; WHO-5, World Health Organization-5 Well-Being Index; WHOQOL-BREF, World Health Organization Quality of Life - abbreviated version; WHQ, Women's Health Questionnaire; XeQoLS, Xerostomia-related Quality of Life Scale; XI, Xerostomia Inventory.
